# The early childhood oral health program: a qualitative study of the perceptions of child and family health nurses in South Western Sydney, Australia

**DOI:** 10.1186/s12903-016-0213-0

**Published:** 2016-05-16

**Authors:** Maxine Veale, Shilpi Ajwani, Maree Johnson, Linda Nash, Tiffany Patterson, Ajesh George

**Affiliations:** School of Nursing and Midwifery, Western Sydney University, Sydney, Australia; Sydney Local Health District Oral Health Services/ Sydney Dental Hospital/ University of Sydney, Sydney, Australia; Faculty of Health Sciences, Australian Catholic University/Ingham Institute Applied Medical Research, Sydney, Australia; Marrickville Community Oral Health Clinic, Sydney Local Health District Oral Health Services, Sydney, Australia; Centre for Applied Nursing Research, Western Sydney University/ South Western Sydney Local Health District/ Ingham Institute Applied Medical Research , Sydney, Australia; Collaboration for Oral Health Outcomes, Research, Translation and Evaluation (COHORTE) Research Group/Western Sydney University/South Western Sydney Local Health District Oral Health Services/University of Sydney/Ingham Institute Applied Medical Research, Sydney, Australia

**Keywords:** Early childhood, Child and family health nurses, Oral health, Qualitative study, Postnatal

## Abstract

**Background:**

Early childhood caries affects nearly half the population of Australian children aged 5 years and has the potential to negatively impact their growth and development. To address this issue, an Early Childhood Oral Health (ECOH) program, facilitated by Child and Family Health Nurses (CFHNs), commenced in 2007 in New South Wales, Australia. This study builds on the previous evaluation of the program. It aims to explore the perceptions of CFHNs regarding the implementation of the ECOH program in South Western Sydney and the challenges and barriers related to its sustainability.

**Methods:**

A descriptive qualitative design was used in this study. Two focus groups were conducted with 22 CFHNs who were sampled from two Community Health Centres in South Western Sydney, Australia. Data were transcribed verbatim and thematic analysis was undertaken.

**Results:**

Most CFHNs acknowledged the importance of early childhood oral health promotion and were providing education, oral assessments and referrals during child health checks. Many stressed the need for collaboration with other health professionals to help broaden the scope of the program. Some barriers to implementing the program included confusion regarding the correct referral process, limited feedback from dental services and the lack of oral health awareness among parents.

**Conclusion:**

The study findings suggest that the ECOH program is being sustained and effectively implemented into practice by CFHNs. Improvement in the referral and feedback process as well as enhancing parental knowledge of the importance of infant and child oral health could further strengthen the effectiveness of the program. Expanding oral health education opportunities into general practice is advocated, while regular on-line training for CFHNs is preferred. Future research should include strategies to reduce non-attendances, and an assessment of the impact on the prevalence of childhood caries of the ECOH program.

**Electronic supplementary material:**

The online version of this article (doi:10.1186/s12903-016-0213-0) contains supplementary material, which is available to authorized users.

## Background

Early Childhood Caries (ECC) is the most common chronic disease among young children despite it being a preventable condition [1, 2]. ECC is prevalent in both developing and industrialised nations highlighting it as a serious public health concern [3]. Although the prevalence of ECC in some European countries is considered to be declining (1–32 %), rates of ECC in developing nations such as Africa and South East Asia are thought to be approaching epidemic proportions (44–82.5 %) [3, 4]. Western countries such as Canada, the USA, and eastern European nations are also experiencing a gradual rise in cases of ECC. Disadvantaged populations such as indigenous children in Canada (80–90 %) demonstrate very high prevalence rates [3–5].

Almost half (48 %) of Australian children aged 5 years’ experience childhood caries, with rates highest among families from socioeconomically disadvantaged backgrounds [6]. Early Childhood Caries (ECC) are characterised by the presence of one or more decayed, missing (caries related), or filled tooth surfaces in any primary tooth in children between the ages of 0 and 5 years [7]. Untreated ECC causes pain and discomfort which can lead to altered eating and sleeping habits, acute and chronic infections, learning difficulties, impaired speech and even hospitalisation all which have a negative impact on the child’s quality of life [3, 7, 8]. This condition can also have an adverse effect on the quality of life of parents [9].

To address ECC, there has been a heightened awareness internationally to involve child health professionals such as child and family health nurses (CFHNs) in this area as they are in optimal positions to provide screening and oral health education to parents, carers and children [10]. A study in the United States [11] showed that implementing a practice-based oral health intervention using registered nurses and nurse practitioners was associated with overall greater health provider knowledge, and a significantly decreased incidence of ECC. These findings are supported by a recent review of ECC prevention and nursing interventions which showed that some of the most effective methods for reducing ECC included paediatric nurse interventions [12]. The specific aspects of the interventions included: educating parents, examining children’s teeth from as early as 6 months and referring at-risk children to dental services [12].

Australian oral health policy also supports these international initiatives with the belief that oral health promotion for infants and children should be provided by child health care professionals such as CFHNs in addition to and in partnership with oral health professionals [6, 13]. In response to increasing rates of ECC, the Early Childhood Oral Health (ECOH) program was launched in 2007 using a model of shared responsibility facilitated through the use of the NSW Personal Health Record [13]. This program provided parents and health professionals with increased access to health information to enable early identification of the disease and referral of affected children to appropriate oral health treatment services [14].

Key to the success of this program was the early assessment and intervention by child health professionals such as CFHNs. This program provided an opportunity to identify children at high risk of developing ECC, those already affected, and, where appropriate, refer these children to publicly funded oral health care services. Given that there was and remains limited education on oral assessment within undergraduate or post-graduate nursing or midwifery education, educational support for these CFHNs was provided through a program known as ‘Lift the Lip’ [15]. At the time of writing, early childhood oral health is included as an assessable clinical skill within the Professional Practice Framework for CFHNs [16]. The first evaluation of the ECOH program in 2010 [14] identified that parents, as a consequence, had increased access to oral health information and also that CFHNs were incorporating the screening and referral of children to dental services into their practice. However, one of the recommendations of this evaluation was to continue reviewing the program and identifying areas for further improvement [14].

This study now builds on previous work [14] by exploring the sustainability of the ECOH program from a CFHNs perspective in South Western Sydney. We seek to gain an insight into the perceptions of CFHNs in relation to the program, as well as identifying challenges and barriers CFHNs have faced since its inception. South Western Sydney was chosen for this study as it is characterised by families from a lower socioeconomic status and an increased number of families from culturally and linguistically diverse backgrounds (73 %) [17]. South Western Sydney also represents the largest and fastest growing population in NSW, with reported high levels of unemployment and welfare-dependant families [17] as well as high prevalence rates of ECC [18].

## Methods

### Aim

The aim of this study was to explore the perceptions of CFHNs regarding the implementation of the ECOH program in South Western Sydney and the challenges and barriers related to its sustainability.

### Design

A descriptive qualitative approach involving focus groups with child and family health nurses was used to address the aim of this research.

### Participants and recruitment

The settings for this study were two metropolitan Community Health Centres in South Western Sydney, Australia. South Western Sydney Local Health District (SWSLHD) consists of 5 regions and includes 18 Early childhood/ community centres. Ethics approval to conduct this study was granted by South Western Sydney Local Health District Human Research Ethics Committee (HREC/12/LPool/184). Participation was voluntary and privacy and confidentiality of all study information was maintained. Written informed consent was obtained from all participants prior to data collection.

Participants had to be familiar with the ECOH program and providing services to the population of South Western Sydney. Flyers advertising the study along with information sheets were distributed, via nurse managers, to all CFHNs working in the two community health centres . As part of current guidelines, all CFHNs receive training on the ECOH program and are required to implement the program during standard care [15]. The two centres (one large and one small) housed the majority of child and family health nurses that provided care to families in South Western Sydney. Interested participants were asked to contact the project team for further information and were invited to attend one of the focus groups. Twenty-two CFHNs were recruited for the two focus groups −16 from the large centre (16/34; 40.5 % response rate) and 6 from the small centre (6/11; 55.5 % response rate). To maintain confidentiality each participant was assigned a pseudonym based on the study site they belonged to. For example, (N1.1) refers to nurse 1 from the large centre (1) and (N1.2) refers to nurse 1 from the small centre (2).

Participants had a mean age of 50.3 years (SD 9.25) and an average of 10.8 (SD 7.4) years of experience with most having a post graduate qualification (72.7 %) (Table 1). At present there is no specific data on the demographics of CFHNs in NSW, Australia. National data states that the average age for nurses and midwives in 2013 was 45 years however, the proportion of nurses and midwives aged 50 years and over rose from 36.3 to 39.3 % between 2009 and 2013 [19].

**Table 1:** Demographic details of the focus group participants (*N* = 22)

Characteristics	*n* (%)
Age (years)^a^	
30–39	3 (13.6)
40–49	5 (22.7)
50+	13 (58.8)
Highest education qualification	
Bachelors	6 (27.3)
Post Graduate Diploma	10 (45.5)
Masters	6 (27.3)
Nursing Experience (years)	
1–9	10 (45.3)
10–19	8 (36.1)
20–29	4 (18.1)

^a^Missing value (*n* = 1)

### Data collection

Two focus groups were conducted at the community health centres at a time and place suitable to all participants. The focus groups were two hours long and were digitally recorded. Each of the two focus groups was conducted by one lead and one supporting researcher who were trained in qualitative techniques. The focus groups were conversational in nature and topics were established to maintain the flow of the discussion, although participants were not restricted to these specific discussion points. The main topics addressed were the importance of infant oral health, role of CFHNs in providing oral health assessments and referrals to infants, perceptions of CFHNs about the ECOH program and challenges and barriers in implementing the ECOH program.

During the focus groups it was important to maintain good interaction among participants. For CFHNs this was easily achieved as they are a very cohesive group with very little turnover and so most participants were comfortable in sharing their experiences. Further, the Nursing Unit Managers at the study sites were very supportive of staff stating their point of view. A number of strategies were also employed to ensure good interaction in the focus groups such as prompting, checking all participants had voiced their opinions, repeating questions to ensure everyone had an opportunity to speak and also clarifying responses with participants [20]. The smaller group did pose some challenges in terms of interaction and so to address this we purposefully directed questions at all individuals.

### Data analysis

The recorded data from the focus groups were professionally transcribed and uploaded into the qualitative analysis software package NVivo (Version 10) [21]. Initially, transcripts were read repeatedly to familiarise the research staff with the emerging themes. Thematic analysis [22] was undertaken and data were coded into themes and sub themes that depicted the views and perceptions of the participants. Coding was done independently by two study investigators and a research assistant who then compared, contrasted and grouped themes until an agreed list was generated. This list was then circulated to all the study investigators and through a consensus meeting the final coding structure was formed. When consensus was not reached on certain themes and subthemes a third independent coder was utilised. This process added rigour to the analysis through peer coding. Confidentiality and anonymity was assured by using numbers and letters for participants when identifying statements from individual CFHNs across both study sites.

### Trustworthiness

Data analysis and the establishment of themes and subthemes was undertaken using three members of the research team who independently analysed the data. A meeting was also held with team members to ensure consensus. Use of qualitative analysis programs such as NVivo, as used here, also supported the credibility of the analysis [23]. Furthermore, an audit trail [24] documented the coding decisions as they progressed in the analysis.

## Results

The main themes that emerged focused on the perceptions and challenges of implementing the ECOH program (Table 2).

**Table 2:** Themes and subthemes emerging from the findings

Themes	Subthemes
Perceptions of the early childhood oral health program	• Importance of oral health promotion • The need for collaboration • Training and resources
Challenges in implementing the ECOH program	• Issues with referral process and feedback • Lack of awareness and misconceptions among parents • Scope of the ECOH program

### Perceptions of the early childhood oral health program

#### Importance of oral health promotion

Most CFHNs acknowledged the essential role they played in promoting early childhood oral health and felt it was an integral part of childhood development:

*We promote infant health right from the word go, so the moment the teeth start to erupt we’re looking at dental hygiene right from the very start…*(N8.1).

*Dental hygiene is as important as weight and height and growth and development. It just all goes hand in hand…* (N5.1).

The participants also highlighted the importance of educating parents about the correct practices to minimise the risk of ECC. They displayed a solid knowledge base in this area and were confident in communicating this information to parents during their post-natal visits. The information conveyed included the following:

*Promoting water through a sippy cup, at the 6 month mark getting rid of bottles at 12 months. Minimising the use of dummies and then the nutrition starts..*(N3.1).

*Encouraging them to introduce solids around 6 months of age but at that point I will talk about oral health. Try to educate them on nutritious foods and drinking water only…*(N7.1).

The CFHNs stressed the importance of encouraging parents to take an active role in conducting oral health checks on their children and to *‘do it on a regular basis … when they have the opportunity every day’*(N2.1) as well as ‘*read the blue book (*Australian Personal Healthcare Record for Children) *so they are well aware of the information…* (N8.2). This view on the importance of early childhood oral health was reflected by a majority of CFHNs who also agreed that their efforts were making a positive impact on the families. As one nurse commented ‘*when they have gone to the dental clinic and come back they’ve all had a very positive experience…the children are actually excited about brushing their teeth…it’s a very positive experience I think for the whole family’* (N6.1).

#### The need for collaboration

Many participants stressed the need for collaboration with other health professionals to help broaden the ECOH program and access more children:

*‘I think it’s an ideal opportunity* (to promote early childhood oral health) *in day care centres, antenatal clinics…preschools. I think the GPs and medical centres should also be pushing this. They all turn up to the doctors at some point.’* (N4.2).

Using services that are also more commonly visited by parents, such as the chemist (pharmacist) and General Practitioner (GP), was discussed by the participants. It was perceived by CFHNs that caregivers and parents were more likely to attend these services for general health consultations and specific services such as immunisation. One CFHN also suggested that it would be ‘*worthwhile introducing dental checks as part of the normal personal health record book’* (N3.1). ‘*It doesn't necessarily guarantee that they'll do it but …you know most mothers are aware of the Blue Book checks’ (*Australian Personal Healthcare Record for Children) (N2.1) and this would mean that all parents would have to take their child at some point to visit a dental service. This would be especially beneficial for those children who don’t visit the community and family health centres regularly as they would still receive a dental check-up at some time during their preschool years.

#### Training and resources

The majority of CFHNs were satisfied with the current training provided as part of the ECOH program. Most received training when they commenced employment with their respective community health centre and after that were provided additional training on request.

*‘We have been trained in nutrition and oral health and all that sort of thing … I think we’re pretty well equipped to answer any questions…’*(N5.1).

Most participants were also satisfied with the available ECOH resources and felt the program provided a straight-forward explanation of how to assess a child’s teeth (referring to the ‘Lift the Lip’ procedure) [15].

‘*you just lift the lip and have a look at it… if you’re not sure, just refer. So it’s not a drama’.* (N2.1).

*Lift the Lip, the brochure itself is a good resource. It’s got the information there, it’s got photographs to refer to. I’m not sure that I would use anything other than the Lift the Lip brochure anyway. I think it’s very self-explanatory* (N1.1).

However, some CFHNs suggested that the ECOH training could be reviewed ‘*every year’* (N9.2) in order to address confusion regarding the referral process and deliver new updates or changes to the ECOH program thus ensuring the currency of evidence based practice. Another nurse also stated that the resources could be modified to include more realistic images of poor oral health frequently observed by the CFHNs to motivate parents to be more proactive in this area.

*You could put some worse photos on there. Sometimes the photos - I don’t think the photos on Lift the Lip are as bad as what you see out there sometimes. I think you could put some really dreadful ones on and I think it might motivate the parents a bit more* (N6.1).

### Challenges in implementing the ECOH program

There were a few challenges highlighted that impacted on the CFHNs ability to implement the ECOH program.

#### Issues with the referral process and feedback from dental services

One concern raised by some CFHNs related to the referral process to the public dental service. (Currently for the ECOH program, urgent dental referrals can be telephoned through or in non-urgent cases a paper form can be faxed). The issues experienced by the participants focused around the appropriate referral process to follow for children to receive prompt treatment. For example:

*I think there was some confusion particularly initially and I found lots of people were just saying ring the number. Not realising that you don’t get the same response from the number and by faxing …* (N10.1).

*There’s been a bit of confusion over the years I must admit, I’ve had to come back and ask what’s the latest pathway because I don’t think we’ve had definite referral pathways…* (N1.1).

Feedback from the dental services on the status of the referred child and whether he/she did seek care was another issue highlighted, with one participant reporting that she ‘…*didn’t get any personal feedback…[from dental services]’* (N6.1). The majority of CFHNs agreed that feedback from the dentists was useful for them, especially if the family did not attend the clinic, so that they could follow up.

*The feedback that they didn’t attend would be very helpful because I think I would feel the onus is on me then to chase them up and get them to go’* (to the dental services) (N3.1)

*Even if it’s* [feedback] *just an email. I wouldn’t mind an email; I think that would be great* (N6.1)

However, one CFHN was happy with the current process:

*Well there’s an oral health referral form and its filled out, you fax it and they’ve called back within a week or two* (to make an appointment)*, it’s very good.’*(N15.1)

#### Lack of awareness and misconceptions among parents

Another concern among participants was the misconceptions and lack of awareness about the importance of early childhood oral health among parents. Many CFHNs felt that parents believed deciduous teeth were not important as ‘*it’s their first teeth … it’s not going to affect them later.*.’(N1.2) or ‘*That tooth will come out and the next tooth will be beautiful…*’(N2.1). Parents generally had *‘a perception out there that any mouth … can be solved… it’s not about preventative…there is a perception out there that any problem can be fixed with your teeth.’* (N3.1).

CFHNs were particularly concerned about inappropriate feeding practices and oral hygiene habits that were being followed by parents, all of which increased the risk for the child developing ECC. Numerous examples were cited:

*One couple has come into mind at the moment, where the child wouldn’t eat anything but chocolate… Apparently the father had introduced the child to chocolate and didn’t see any problem with that…* (N4.2).

*We have a baby whose family are all carnival workers… he lives on fairy floss and hard lollipops…I’m trying really hard to get a fruit bowl in the fridge as an alternative but grapes don’t seem to cut it the same way lollipops do …* (N3.1).

*I can’t believe how many people put their children to bed with a bottle of milk,* (N4.1) *yes it a big problem in this area…* (N5.1)*, Or stick their baby’s dummy in their own mouth before sticking it in the baby’s mouth* (N6.1).

Nevertheless, many CFHNs felt that continuing to promote the ECOH program could raise awareness among parents about the importance of oral health and dispel many of their misconceptions. Numerous suggestions were also proposed to further assist in the promotion of the ECOH program such as advertising on ‘*shopper dockets’(Free or reduced item cost opportunities detailed on the back of shopping bills)* and through ‘*Centrelink (Centre for unemployment or disability pensions) payments… so they know the program is out there*’ (N7.1) *‘I think it’s just general reinforcement, that they’re getting the same message everywhere they go…’* (N4.1).

The participants also suggested a variety of ideas that could appeal to parents and children about oral health such as ‘*Free toothbrushes for kids… One toothbrush to each baby I reckon’* (N11.1) ‘*Definitely’* (N12.1).

Another suggested:

*I think if you just had a five minute DVD on dental health for babies and for children that we could run over and over, out here that would be an ideal opportunity. That’s my point of view because you get such a cross section of people, antenatal, the whole lot…’* (N9.2)

#### Scope of the ECOH program

Although there was general consensus that the ECOH program was having a positive impact on the health outcomes of families, some CFHNs felt that there were groups within the population that were not being accessed. Only ‘…*the motivated ones’* (N9.1) were seen by CFHNs and, as one participant noted, ‘there’s *a whole section of people that we’re actually not seeing…’*(N2.1). This was reiterated by another participant:

*‘I think it’s really important to remember what everyone else is saying, that the ones we really need to see the most are… the bottom sort of 10 per cent. They’re the ones we need to find…’* (N1.1).

Another issue was the reduced presentation of caregivers and children to the clinics due to various factors including families consulting with other health professionals ‘*they may not come and see us but they will turn to a doctor when they’re not well’* (N7.1) and parents returning to employment earlier:

‘*… so someone might follow through for the first year, then they’ll go back to work and we won’t see them again until the four year check. You can get mums going back* (to work) *after 3 months and then you just don’t see the children anymore’.* (N4.2).

Finally, the participants also expressed concern that the ECOH program may have limited efficacy in relation to caregivers from a lower socioeconomic status, with lengthy waiting lists to access public dental services and extended waiting times on the telephone when trying to make dental appointments using the health district’s call centre number (1300 number). They believed that the cost incurred when using a mobile telephone and waiting to connect to the call centre was ‘*making it hard for them to get into the system’* and they are doing them a *‘disservice’* (N4.1) which may be a deterrent for parents in facilitating their children’s oral health.

*We’re asking them to call into that 1300 number [call centre number to make dental appointments in the district] which is unlimited (in terms of wait time and call charges) once you’re on there. Now I’ve had girls [referring to mothers of children] tell me that they used all their credit [referring to mobile phone call credit]… now I don’t think that’s fair when we’re trying to target vulnerable people....We’ve actually made the service very difficult to get hold of… they just can’t afford to spend a lot of time on the phone, they can’t afford to ring and make an appointment…’*(N9.1).

The nurses also discussed that the financial constraints of parents and carers accessing private dental services for themselves, could influence their uptake of the ECOH program for their children

*My ‘beef’* [concern] *is* [that] *not all adults can afford to go see a dentist. It’s very expensive to go see a dentist if you have a limited income. If a parent doesn’t see a dentist even for normal preventative cleaning and checks then there’s no way they’re going to take the children along.* (N10.1).

## Discussion

The focus of this study was on exploring the perceptions of CFHNs towards the Early Childhood Oral Health program in South Western Sydney to identify enabling factors, and challenges and barriers to its sustainability. The ECOH program supported the new and innovative role of CFHNs undertaking oral health assessments in children. Several studies have acknowledged that there is an increasing role for other health professionals in supporting oral health [12, 25] and this study along with the previous evaluation of the ECOH program [14] have confirmed the feasibility of such a role for CFHNs. Increasingly, nurses and midwives are expanding their scope of practice to include oral health promotion for pregnant women and their infants [26].

From the findings, it is evident that CFHNs were well aware of the importance of childhood oral health and the effect that ECC could potentially have on the growth and development of children. The nurses felt confident and took ownership of their role of incorporating oral health promotion into practice and referring children to dental services, even going to the extent of identifying ECC risks in other siblings and family members. This finding is in contrast to an earlier study [27] that suggested Maternal and Child Health Nurses (MCHNs) (equivalent to CFHNs) may not have the confidence to assume such a role in oral health promotion. This lack of confidence in assessing ECC has been reported in other health professionals as well [1, 28, 29] and appears to be primarily linked to lack of training and education. The sustainability of the program 5 years on has been assured with effective oral health education, assessment and referral of children to appropriate dental services being facilitated by CFHNs. However, one of the limitations of the ECOH program to-date is that it has not been evaluated in terms of its effectiveness in reducing early childhood decay and this is an aspect that needs to be addressed in future studies.

Within the positive statements, some challenges were identified by CFHNs during the implementation of the program, the most significant being the lack of awareness among parents and caregivers about ECC. This finding is consistent with an earlier study [30] which showed discrepancies in the levels of understanding among parents and caregivers in relation to oral health. These findings underscore the importance of implementing oral health promotion strategies during the post-natal period. This critical period of the child’s life can be largely influenced by the attitude of parents and carers to the child’s oral health practices [25, 27].

The ECOH program delivers oral health information, education and support through written resources and contact with child health professionals [14]. However, given parents’ lack of awareness of the importance of oral health to the child’s growth and development [27], additional strategies may be required to target vulnerable populations. The participants suggested that information campaigns within general practices or antenatal clinics may be an appropriate setting to further promote oral health. Also participants proposed providing free toothbrushes and/or oral health promotion educational material to be provided at locations such as centres where vulnerable populations often frequented such as ‘Centrelink’, where pensions relating to unemployment or disability are presented.

Another challenge that emerged from the findings was the variation in the CFHNs experience of the referral and feedback process. In particular, misunderstandings in relation to the referral process and feedback to the CHFHs presented difficulties for them in their implementation of the ECOH program. This issue is a new finding that was not raised in the previous evaluation [14]. Current training procedures need to be revised to ensure clarity regarding the referral process, including providing timely feedback to CFHNs on parents and children who did not attend scheduled appointments at dental services.

Providing regular feedback can enable nurses to follow up with those parents who have missed their appointments, help reassure nurses that their interventions are having a positive impact on the children, and encourage nurses to continue implementing the ECOH program. Nonetheless, all dental therapists and oral hygiene therapists in NSW public dental services are required to send a response letter after referral. The issue of feedback from referrals in clinical practice is not unique to CFHNs or this study, with failures in communication between health care providers leading to the breakdown of continuity of care and delayed diagnosis [31]. Communication (including feedback) between health care providers remains a vital component in the success of referrals.

Numerous studies focusing on non-attendance at health services across a range of discipline areas have been previously conducted and much is known about the contributing factors, especially in relation to patient behaviours and motivation [32, 33]. Although complex and wide-ranging, some common determinants of non-attendance include length of waiting time to appointment, communication or service failures, and forgetting. Prompts are regularly used by many health professionals such as text messaging patients 24 h prior to attending [34]. This approach and other strategies to improve non-attendance is a key area for future research.

A number of suggestions were put forward to further complement the ECOH program. These included: developing more oral health promotional resources such as a DVD for Community Health Centres, and adding a dental visit to the health record. Such a DVD could assist in increasing the awareness of parents, carers and the general population in relation to ECC. This view is similar to Silk’s [35] suggestion that visual promotional media should be used in the waiting rooms of medical practices to extend the exposure of patients to oral health messages. Use of the DVD in various contexts such as Doctor’s surgeries, dental clinics, and community health centres would reach a broader audience, potentially increasing awareness of ECC and providing pathways for access. The existing Lift the lip brochure being used in the ECOH program [13] could also be revised to include more graphic images depicting early childhood caries which may help motivate parents to be more proactive in regards to their children’s oral health. This is especially true considering graphic images have been shown to be an effective tool in raising awareness and changing behaviours/habits in other health related areas such as smoking [36].

The ECOH training was another area that could be improved. Currently, training is provided to CFHNs on request by the ECOH co-ordinator through face-face workshops. Our findings suggest that CFHNs would prefer to seek training on an annual basis, rather than being restricted to the commencement of employment. This would ensure that all nurses were provided with updates on the program and that their practice was underpinned by the latest evidence in this area. Although not mentioned in the findings, the medium of training could also be changed to an online program which would provide CFHNs with the flexibility to access training at any time or place convenient without needing to rely on the ECOH coordinator. This would also help expand the scope of the ECOH program to rural areas without any additional resources required. Currently, online training programs in oral health have been used successfully in Australia with other health professionals (midwives) [37, 38] and are an option that should be considered for the ECOH program in the near future. An online program previously existed for doctors through the Royal Australian College of General Practitioners but is now no longer available.

Lastly, the inclusion of general practitioners, practice nurses or nurse practitioners within the ECOH program was highlighted as an important strategy to improve the effectiveness and reach of health promotion initiatives. This was especially important as after 12 months there is a general decrease in the attendance rates of parents/caregivers and their children to community health centres, thereby limiting the scope of the CFHNs [35]. Further, as parents are more likely to consult with their GP in relation to their child’s health, especially given the Australian Personal Health Care Record for Children (Blue Book) recommendation for vaccinations [39], doctors are well positioned to continue on from CFHNs in providing oral health promotion to infants and children. [40]. This inclusion of GPs (or practice nurses or nurse practitioners in general practice) in the promotion, assessment, and referral of children with ECC is well supported in the literature [34, 41, 42] and is advocated in the ECOH program guidelines [13]. However, there is limited information on whether GPs or other health professionals have adopted the ECOH program successfully and further research is this area is recommended.

### Limitations

Although not the focus of this study, one of the limitations is that neither the initial evaluation of the ECOH program [14] nor this qualitative study has examined the effectiveness of the ECOH program in reducing ECC rates in children. One of the challenges completing an evaluation of this kind is the variation in the uptake of the ECOH program among various local health districts in the state making it difficult to do targeted analysis using population data. Secondly, only two focus groups were conducted in this study and so data saturation may not have been achieved and the findings may not fully represent CFHNs in the health district. Another limitation is that the setting for this study was in an area characterised by a large population of families from culturally and linguistically diverse and socioeconomically disadvantaged backgrounds. Hence, the findings may not reflect all children and health services within NSW, particularly those from more advantaged communities.

## Conclusion

This study suggests that CFHNs have successfully increased their scope of practice and readily adopted the ECOH program providing vital oral health education, assessments and referrals to children in South Western Sydney. Some challenges have been identified that need to be further explored including broadening the scope of the program to include general practice. Information campaigns within general practices or antenatal clinics may be an appropriate setting to further promote oral health In addition, improvements in referral and feedback processes as well as enhanced parental knowledge of the importance of infant oral health could further strengthen the effectiveness of the program. Approaches to managing non-attendance require further investigation. Nevertheless it is clear that the ECOH program is being translated into practice and the next step should be to undertake a comprehensive evaluation to assess whether there is any improvement in the ECC rates of children as a result of the implementation of this program.

## Ethical approval and consent to participate

Ethics approval to conduct this study was granted by South Western Sydney Local Health District Human Research Ethics Committee (HREC/12/LPool/184). Participation was voluntary and privacy and confidentiality of all study information was maintained. Written informed consent was obtained from all participants prior to data collection.

## Consent for publication

Not Applicable.

## Availability of data and materials

The dataset supporting the conclusions of this article is included within the article as an Additional files 1 and 2.

## Additional files

Additional file 1: Transcript 1. (PDF 195 kb) Additional file 2: Transcript 2. (PDF 282 kb)

## Abbreviations

CFHNs: child and family health nurses
ECC: early childhood caries
ECOH: early childhood oral health
