# Supplementary material for: The early childhood oral health program: a qualitative study of the perceptions of child and family health nurses in South Western Sydney, Australia
Source: BMC Oral Health. 2016 May 16;16:56. doi: 10.1186/s12903-016-0213-0 (PMC4867529; doi:10.1186/s12903-016-0213-0)
Supplement: Additional file 1: — Transcript 1. (PDF 195 kb) [file 12903_2016_213_MOESM1_ESM.pdf]

Transcript 1

Facilitator 1: Just a few housekeeping rules with focus groups. I mean you guys are probably used to it but because we're recording we would like everyone to be able to be heard, especially - so hope - would like that no one - gives everyone a chance to speak. So if everyone's speaking together it's difficult for us to actually - when you go through the interviews - analyse it. So maybe we'll wait till one person finishes and then we'll start the next one. If at any point in time you feel that this is not going the right way then we can stop the recording and go from there.

Female: Can I just ask a question? They're getting into the first [unclear] very quickly, the longer ones that I've referred, they're not - the prevention ones, the ones that actually need [unclear]…

Facilitator 2: Yep.

Female: …[unclear] getting referred to *** Hospital but…

[Over speaking]

Facilitator 2: *** Hospital.

Female: Yeah. There's a big waiting list and then people move on and I think that's where they…

Facilitator 2: I think with young children it's very hard to do treatment in the chair because they don't stay. They are nervous and it's very hard. So either we have to give them sedation, the nitrous oxide, to calm them down, or they have to go under general anaesthetic. Now to go under general anaesthetic, we do have a few hospitals but we have only two sessions we can do a week, so therefore the waiting time increases. With nitrous oxide now more and more clinics are doing this. So if the child can be managed in a chair with the help of nitrous oxide it's not a problem. It's only when they have to go under a general anaesthetic that the waiting time becomes long.

We are just bound by how much funding we get to get sessions at the hospital, that's where we get stuck. We would like to - we have people staff trained to go and do general anaesthetic sessions…

Female: That's what happens in all the [unclear].

Facilitator 2: Yeah. So that's where we have limitation. But if the child can be managed, we have to pull out a tooth and the child is around four years and can probably be managed, we do it then and there. It doesn't take long. It's only when we can't do that, yeah.

Female: What's the current waiting list like for…

Facilitator 2: There is no waiting list for child.

Female: Not for - for *** Hospital?

Facilitator 2: Again *** Hospital only if you have to be seen by a specialist and all be seen under general anaesthetic. I think the times [unclear]… is about six months to a year, that's my understanding. That's why we want to do this ECOH program because we can prevent many from reaching that stage. That's why a lot of emphasis is being given on ECOH and I think it's a fantastic, brilliant program. If we can get referrals early on we can just manage them by preventing the [diseases] … giving mothers the information, so that's what we call … more and more people coming to us.

Female: How close are we to having Medicare cover regular checks for dental prevention - dental problem prevention? Are we close to having that?

Facilitator 2: For adults?

Female: For children and adults.

Facilitator 2: Children don't have to. All children under the age of 18 are covered free of cost in public sector.

Female: But there's - often there can be a wait in those places just to book in for a regular appointment for preventative dental health.

Facilitator 2: Yes. Most of the treatments are done very shortly. If there is a regular annual check-up then they go on a waiting list. We're getting

more and more funding now from the Government to get people off those waiting lists, so the times are reducing…

Female: Is there no means testing?

Facilitator 2: Not for children.

Female: Oh, because I was thinking for here that the mother had to have a Health Care Card or…

Facilitator 2: For children I think that's excluded. For adults yes, you have to have a Health Care Card.

Female: No I thought even for the child.

Facilitator 2: If they have a Medicare card, a child, I think they become [unclear].

Female: I think many parents have been asked to produce a Health Care Card for their children in order to receive a service. I know that…

Facilitator 2: For specialist services yes. If they have to be seen by a specialist then they need to have it. Because we have so many - and less resources, the priority is given to children with Health Care Cards, yes.

Facilitator 1: So I just wanted to highlight that when we go through these focus areas, please give as much examples as you like from your own experiences. Everything you say here is confidential so if you by accident give some sort of identifying in a statement that's alright, it will be deleted from the audio.

So I'm going to start off by basically going through the focus areas. While that's going on we're just going to send this around. It's just basically three questions, basic descriptors, you know your highest qualification, how many years you're working in the child and family health business.

So the first thing I'd like to talk about is the importance of oral health for children. So do you think it is an important aspect for infants?

Female: Yes.

Facilitator 1: Okay and so there's unanimous agreement. Why do you think it's important?

Female: Well we promote infant health right from the word go, so the moment the teeth start to erupt we're looking at dental hygiene right from the very start. It's good for obviously long-term dental health but that has also cardiac implications and just general health implications.

Facilitator 1: Any other…

Female: Obviously we don't want a child to be in pain and all the problems that creates for the family. Obviously it's a very unsatisfactory situation.

Facilitator 1: So do you think you as child and family health nurses have a role in promoting oral health?

Female: Definitely.

Facilitator 1: So keeping aside this program, in your own words what do you think your role should be basically?

Female: The prevention of [unclear] right from the word go.

Facilitator 1: So when you say prevention, what aspects would you want to I guess…

Female: Sort of like promoting water through a sippy cup, H_20, at the six month mark and getting rid of bottles at 12 months. Minimising the use of dummies and then nutrition starts you know…

Female: Oral hygiene…

Female: But just you know even in regards to nutrition, just even having a textured diet instead of staying on the liquid diet and speech and all that type of thing comes…

Facilitator 2: Someone mentioned oral hygiene. Would you like to elaborate?

Female: Well when the teeth start to erupt, we're looking say from six months on, so just gentle cleansing of the gums using washcloths or small baby dental brushes or - you know the gum massages, those sorts of things? So it starts right from the very beginning and then graduating them up to daily - at least daily or twice daily dental hygiene. So as *** said, it will be water drinking and cutting back on bottles and sticky, sweet foods and things like that.

Female: It's just educating them. I can't believe how many people put their children to bed with a bottle of milk.

Female: Yes, big problem in this area.

Female: Or stick their baby's dummy in their own mouth before sticking…

Female: Yeah.

Female: …it in the baby's mouth.

Facilitator 1: So I'd like you to think about the current practice, or the ECOH program more specifically. So we're just trying to gauge what your understanding is of the ECOH program. So what do you think your role as a child and family health nurse is supposed to be, as per the ECOH guidelines? What do you think you're supposed to be doing?

Female: Identify them and refer them.

Female: So we start with the Lift the Lip protocol according to the personal health record and so checking their mouth every personal health record check and getting the mums to check in between. We give them the flyer [unclear]… put on fridge magnet thing so that they are observant of this white line around the tooth.

Female: It becomes part of the regular checks. So when the child and family checks are done that includes a dental check. Or even a dental reminder to take them to the dentist or have their teeth checked when the parents feel that is appropriate. I think that varies. I don't know about you but I think most of the children I usually refer to have a dental check around about three. I don't know what everybody else does.

Female: So by the time you turn three, yeah.

Facilitator 1: So we're just trying to see what a typical visit would be like, where you would incorporate your oral health. So you know if you could just explain exactly when you do it, like in a typical [unclear]?

Female: Well we have the Blue Book. We have the standard Blue Book checks which we call them the PHR - personal health record checks.

So they're done at six months, 12 months, 18 months, two years, three years, four - that type of thing. So that's where the - it's a definite tick box that you have lifted the lip and discussed oral health and hygiene. You know informing them not to give them toothpaste prior to 18 months. Then the amount of toothpaste that they need to have and all that sort of thing.

Female: One of the tick boxes for 12 months is does your child have a toothbrush.

Female: But even before that when we start talking about introduction of solids, I tend to talk about it at four months of age. Try to encourage them to introduce solids around six months of age but at that point I will talk about oral health. Try to educate them of nutritious foods and drinking water only. Then sort of giving them the idea that they need to look after - it's not only for obesity but it's also for oral health.

Female: I think too you can't neglect the - even just from the word go, even breast feeding is good for good oral health, you know right from the beginning. Even good antenatal care, you can keep going further and further back. So a good maternity diet for the mothers ensures good strong healthy teeth and bones as well in the infant. So it doesn’t just start with the eruption of teeth.

Female: Well that's right. Even though we tell them to give water with their solids, we make sure they have their 600ml of milk to get their calcium into their diet.

Facilitator 2: Do you have any difficulty with lifting the lip or is there a challenge or…

Female: Yes.

Female: Two year olds.

Female: Do you have any tips actually?

Facilitator 1: Do you always do lift the lip or are there times where you don't do it?

Female: Opportunistic. Sometimes if the kids - you just get them laughing and sometimes you have a quick look at the teeth. Or they'll lie down and

sometimes you'll be sort of checking their head circumference and at the same time you take the opportunity to have a quick look at the teeth as well.

Female: Their eyes as well.

Female: Their eyes, their teeth - you just do it all at once. You've got a limited opportunity, you know one or two seconds sometimes, to do three different things.

Female: You can make it a game with the older kids. [Fun LEGO torches] that they like [unclear] or … the parents and so that they want to copy, or an older sibling or something. Sometimes that helps.

Facilitator 1: So would you say most of the times you would yourself administer the Lift the Lip or would you use the carer or the parent to do it?

Female: We do it.

Female: We do it at each check but I would encourage the mother to do it on a regular basis as well when she's got the opportunity every day to.

Facilitator 1: So you're comfortable actually doing the process of Lift the Lip using your hands and doing…

Female: Yeah.

Facilitator 1: There's no problem with that…

Facilitator 2: You've not been bitten by a child…

Female: We always have gloves on when we're checking a child anyway.

Female: I have to admit a large majority of the time you cannot do it because the child is crying and…

Female: Yes.

Facilitator 2: If they're crying they're opening their mouth…

[Over speaking]

Female: …or you get them to do you too.

Facilitator 1: So you role play it.

Female: Yeah.

Facilitator 1: Okay.

Facilitator 2: Is there a family circumstance where you might see the parent's mouth or the siblings' mouth and they [unclear]…

Female: Oh yes.

Female: At the six month checks.

Female: What I will do is encourage the parent because they've been pregnant, they've had the pregnancy, they've been feeding [unclear]… how are your teeth? Got to look after the baby's teeth. So I sort of tend to put the two together. If the mum needs to go to the dentist then I'll refer her to the dental service then. Because the baby's not yet there but her teeth are poor. So the law of genetics in our trade says that maybe the same thing will happen to the next generation.

Facilitator 2: Absolutely, yes.

Female: So I think *** referred five recently in one family. Now I've referred up to three but I've never done five.

Female: Oh it was two families in one [unclear].

Female: It tends to run in runs. So parents have poor oral hygiene, all the kids have poor oral hygiene. The child - the baby we're looking at for the check is almost setting up for poor oral hygiene.

Facilitator 2: Yes, absolutely.

Female: So my idea is get them all in and then the baby will follow. Hopefully not as damaged as the older kids.

Female: I see mothers that are on a methadone program and so many of them don't have any teeth or very, very rotten teeth. Often when we're seeing the new baby they've got ….They have older children so while I'm actually checking the new baby I'm already checking out the three year old, the five year old as well. I usually do [unclear]… a lot of my referrals come from the siblings. So it's a bit opportunistic at that time.

Facilitator 2: The dental therapists do exactly that. Once they get one child on board they try to get the whole family, so they exactly reinforce what you're saying, so that's brilliant.

Facilitator 1: What process do you follow when you have to refer [unclear]? What exactly do you do?

Female: That's been a bit confusing over the years I must admit. I've had to come back and ask what's the latest because I don't think we've had definite referral pathways. I think at this stage where there's a dental number and we call and we make a referral on the dental number. I'm not sure what happens to it after that.

Female: Well there's an oral health referral form and it's filled out, you fax it and they've called back within a week or two, it's very good.

Female: [Unclear]… work with Aboriginal families. So our babies and our mums go to ***.

Facilitator 1: So my understanding is most of your stuff is electronic. Is that right?

Female: Yeah.

Facilitator 1: So you've got this as a - is this electronic or is it…

Female: No.

Female: Paper [unclear].

Facilitator 1: So is that an issue?

Female: No. I think there was some confusion particularly initially and I found a lot of people were saying just ring the number. Not realising that you don't get the same response from the number and that by faxing you get a much more immediate response.

Facilitator 2: So do you know that by faxing you're putting the child through a different referral pathway than a mum about the child? It's out of…

Female: Yes…

[Over speaking]

Facilitator 1: You don't think that? Okay. So there is a difference between actually specifically using the phone than just calling a generic number?

Female: Yes.

Female: They don't answer the generic number. It's very busy.

Facilitator 1: Are you aware of basic public dental service contact details and generally who you would need to contact if you had to ask something or…

Female: Yeah they just don't [unclear].

Facilitator 1: Well I mean let's say you were in part of the community and one of the parents asked you would you…

Female: We've got the [unclear]…

Female: It's very difficult to infiltrate on the phone number. So the unreliable families, if you say look I'll wait here with you and call the number, you'll be still there at dinner time. We don't have the time to do that. So if it's under five I personally will refer it through fax. If it's over five I'll attempt to get the parents to ring it. I explain it's like the old age pension. Once you get it, you've got it for life. So I sort of say to them keep persevering, keep persevering, soon you'll talk to a human, then they'll get you right.

But it is a problem because I feel that the most vulnerable ones have got texting credit or limited phone credit. We're asking them to call into that 1300 number which is unlimited once you're on there. Now I've had girls tell me that they used all their credit up on [unclear]… Now I don't think that's fair when we're trying to target vulnerable people in the modern age of technology. They've all got internet and they've all got email…

Female: But they don't have landlines.

Female: But there's no way they can make a landline call and wait for 20 minutes. They've got texting and they've got email, which is the standard way we all communicate. But with the public that are vulnerable that's too hard. So I think we're doing them a disservice,

making it hard for them to get into the system it's already [unclear] to get on the [unclear].

Facilitator 1: That's a good point.

When you do make a referral, or you fax it, the next time you see the parent do you follow up and ask them well did you guys actually see the dentist or…

Female: Yeah I do.

Female: But see you mightn't see the same people again.

Facilitator 1: So is it documented in the notes?

Female: Yeah.

Female: In the progress notes and you read back over the other notes to be able to follow through.

Facilitator 1: So it's the general practice that you will follow up with the…

Female: [Unclear].

Female: Is there any feedback? I've only referred a couple of families, one was a refugee family that had only been in the country for probably eight or nine weeks, not very long. Both children had terribly rotted teeth so I referred them using the fax actually from memory now. I wasn't sure whether to just refer one child or both children because I think there's room on the list to put siblings as well. I just can't remember what I did. But I didn't get any personal feedback. The child went - I assume went to the dental clinic, was contacted and probably went to the baby health centre sometime after that. I don't know because I wasn't in the area. But I didn't get any personal feedback even though I think my details were taken at the time.

Facilitator 1: So would all of you like to get some sort of feedback…

Female: Even if it's just an email. I wouldn't mind an email, I think that would be great.

Female: I've been getting feedback.

Female: I've had it too.

Female: So I wonder if you only get feedback if they attend. Do they let you know if they don't - are unable to contact them? I don't know.

Female: No.

Facilitator 2: If they don't come to the clinic they're not seen, they become one of [unclear]…

Female: So I'm thinking that's probably happened.

[Over speaking]

Female: Because the ones that have attended I've got feedback.

Facilitator 2: But sometimes the dental clinic will still forget to fax back or the details do not come clearly or something and they don't have the right email address. So that may be an issue as well. But most commonly it might be that they didn't turn up after getting the appointment here.

Female: See the feedback that they didn't attend would be very helpful because I think then I would feel - I feel the onus is on me then to chase them up and get them to go. These children have terrible teeth and it's just the - I mean once they started school that's a real sad situation for these children. They'll be bullied at school the moment they get there.

Female: *** did the people speak English?

Female: Yes they were quite - not bad English speaking. I think it was like Indian or Bangladesh background from memory. It was a new baby I was visiting on a universal health visit but then these two terrible toddlers came out with - lovely beautiful kids but terrible, terrible teeth. It was shocking to see it actually.

Facilitator 2: So do you tell mum or do you have that information that if the baby teeth are rotten then the adult teeth may have the same fate?

Female: Yes.

Facilitator 2: Do you inform the mothers about that?

Female: Yes.

Female: I was quite surprised that they were under the impression that this was just the baby teeth and it wouldn't matter, that they would drop out and then they'll have perfect teeth underneath. It was…

Facilitator 1: Is that a common problem you've got…

Female: Yes.

Facilitator 1: Yeah it's quite common.

Facilitator 2: So the perception that the bacteria still remains in the mouth and doesn't go anywhere and just attacks the new teeth is not sinking. So…

Female: That tooth will come out and the next tooth will be beautiful, that's what they [unclear].

Female: You've got to look at the diet of the child.

Facilitator 2: Of course, it's multifactorial.

Female: I've got a baby whose family are all carnival workers - well he's not a baby any more, he's four. He lives on fairy floss and hard lollipops. Now I [unclear] with that family. So he's got poor dental hygiene already. I've sent his mother to the dental surface [service], I've got to prioritise, there's 14 teeth she's got to have out. But as I keep explaining to her very gently because I see her weekly, that if - I don't want to still keep going to see her weekly for the two years. That if they keep giving him fairy floss every morning when they make fairy floss for the carnivals on the weekends. Then he has free access to all the lollipops and all the sweet things they give out at carnivals, that child's never going to have any teeth.

So I'm really trying hard to get a fruit bowl in the fridge as an alternative but grapes don't seem to cut it the same way as a dozen lollipops do. So my job is to educate two generations of extended family for the third generation this child belongs to. They all live together. So that's fundamental to us as much as good oral care at

home and in the centre, you know [unclear]. It's really hard. That's really, really basic. Don't feed your child sugar all day.

Female: Sometimes you're really up against it. We've got a family that the three year old's had her teeth removed, front four teeth. The two year old's had his teeth removed and it's like okay with this new baby. You know the bottle's going at 12 months and da-de-da. One of the other girls went and saw him the other week and at four months old he's got the dummy stuck in a Paddle Pop. You think it's just not…

[Over speaking]

Female: No, it's just so sad.

Female: But he likes it.

Female: He loves it.

[Over speaking]

Female: Coca-Cola in the bottle.

Female: That goes on to behaviour issues then which means they don't clean their teeth because they're all razzed up from these terrible diets.

Female: But like when you watch the news it's like every time there's a news story on you look at people and so many people have rotten teeth. You know the adults have all got missing teeth, one here or - and I find that just amazing.

Facilitator 1: Do you see Coke in the bottle a common…

Female: Yeah, oh well not common but…

[Over speaking]

Facilitator 1: From time to time?

Female: Or cordial.

[Over speaking]

Female: Or even chocolate flavoured milk.

Facilitator 1: So when you think about - let's go to the ECOH program now. The first thing when you say the ECOH program, what comes to your mind? Like your general perception of the ECOH program.

Female: To be honest we haven't thought of it - well I haven't. If you just out of the blue said ECOH program I would be [unclear].

Female: What is it, yes.

Female: Because it hasn't been referred to in that way.

Facilitator 2: So what do you know it as?

Female: Lift the Lip.

Facilitator 1: So what's your perception - first thing that comes into mind when you talk about the Lift the Lip. What is your general perception of the whole process?

Female: Well it's just [unclear]…

[Over speaking]

Female: [Unclear].

Facilitator 1: Okay so…

Female: I just think of the brochures - you know the brochures that we give out at different stages and then there's a discussion about the brochures when we're giving them out.

Female: Just creating awareness for parents. I'm quite surprised that parents don't think much at all about the kids' teeth. Even those that haven't been to the clinic for ages, I go do you brush your teeth and they go oh do I have to brush their teeth? It just surprises me that they don't think about doing that with their children. So the Lift the Lip certainly is a reminder that they need to do that.

Female: Also the introduction of a cup at six months, they kind of go really?

Facilitator 1: Okay, so that might flow on to the next one I was going to say. So do you feel there are positive aspects of this program and if so what are they?

Female: Oh yeah.

Facilitator 1: What you know…

Female: I think it's just general reinforcement, that they're getting the same message everywhere they go, that dental hygiene is as important as weights and heights and growth and development. It just all goes hand in hand.

Facilitator 1: Any other positive…

Female: The Lift the Lip brochure with the photos on it is great, it really makes a difference.

Facilitator 1: Visually?

Female: Yeah.

Female: They need to see it.

Female: Yeah well see I give out the healthy mouth one as well and then the water in the cup from six months. When I talk about giving the water in the cup for six months I explain that a water bottle is just as good. I'm finding that I'm having more success with water bottles than cups. The thing with the cups too is they're got the valves on them that make them very hard to sip. Now I don't mention any brand because we're not meant to, but there's one particular clothing company that sells cotton clothing. They have a very good water bottle for babies with a child-safe top.

So for $5 they're better off to go and buy that and the baby just [sucking noise] [unclear] back. So it's easier than a beaker.

Female: It's not so good for speech though because we're actually trying to get them [unclear].

[Over speaking]

Female: Yeah I just think they're drinking water [unclear].

Female: The whole idea is to take that thing off them [unclear] to drink from a cup.

Female: That's right, a [wide beaker].

Female: Rather than suck it up.

Female: I think having it written as well to get rid of the bottle at 12 months is…

Female: That's really good.

Female: Suddenly it is - it's down there. Because a lot of mothers I don't think really talk about when - they just keep giving it. [Unclear].

Female: A lot of people are surprised at that.

Female: Yeah, that the child's old enough to not have a bottle, you know oh but they're too little, they still need their milk.

Female: That's the message we need to do, is get rid of bottles at 12 months.

Female: They're not going to be [unclear] a bottle. A lot of them use that…

[Over speaking]

Female: So they just put them down with the bottle and leave it at that.

Facilitator 1: I would love to hear some examples that you can comment where you've seen a positive outcome of the ECOH or Lift the Lip. Has there been any - you know when you go back next time, any positive examples?

Female: I think they do listen. They take their information on - most do take the information on board.

Female: I've been amazed at whose children have the cavities. Because some of the well educated women that have everything that opens and shuts. I don't know whether they feed their children lollies early or what, but I've been surprised at some of them. The mums will have stunning teeth but they don't think that the children will have rotten teeth. Yet you open their mouth and the two year old and the three year old have dental caries. They've been dumbfounded that they've got these caries. You know the late night snacks and having something after dinner after they've brushed their teeth and putting them to bed and all sorts of things.

But yeah I've been surprised at who has the dental caries.

Facilitator 1: Right, okay…

Female: It's not just the lower socioeconomic ones that just don't think about brushing their teeth or do put their babies to bed with bottles.

Facilitator 1: So generally would you say that whenever you go back you've seen a positive outcome of this whole process? Like they've at least tried to heed your advice?

Female: I think so. I think that they need to know and when they know they usually [unclear].

Female: They've all been happy when - the ones that have used - where they've gone to the dental clinic. When they've come back they've all had a very positive experience. Most of the ones I've referred then go on to the *** Hospital. But the first - yeah I think they have - they get given the toothbrush and toothpaste and the child's - they must be very good because the children are actually excited about brushing their teeth and that. So it's actually a very positive experience and educational I think for the whole family.

Female: I think though apart from the people that I've visited with the Sustained Home Visiting program and people that *** sees for example with drug and alcohol. A lot of clients - it's a voluntary service so they only come to us if they're fairly motivated anyway. So I find yeah mums are good. Most of them will say yeah I'm giving my child water now and all that sort of thing. But they're really the ones that - you know they are motivated but we're missing - there's a lot out there in the community we don't see.

But we can only do what we can do so hopefully at some point they've got the information.

Female: They'll see somebody.

Facilitator 1: Yeah, or they can pass on the message maybe to someone else.

Female: Even the ones that we don't - you know that don't need a referral to see the dentist or anything. At least if they're coming to clinic and

every time they come in for a check someone's looking in their mouth at least it's normalising the dental experience. For parents and their kids I suppose.

Facilitator 1: What about any negative aspects of the program - ECOH program? I mean negative aspects that you may have encountered or [unclear].

Facilitator 2: You mentioned both form versus fax so that has come through.

Female: The fact that we don't find out if the client did not attend. I've had a client come back a year later for the next check. The child is older so I only need to see the child yearly. I looked back in our notes and noted oh yes, referred to - I asked if the mother attended. Oh no, no, I didn't get to that one, yeah I probably should ring up again. Whereas I would have hoped that there would have been a bit more follow-up or that I would have notified and then I could have followed it up as well.

Female: I think it would be worthwhile even introducing a dental check as part of the normal PHR book as well. That it became something that every parent had to do with their children at some stage. It doesn't necessarily guarantee that they'll do it but I think it's - you know most mothers are aware of the Blue Book and the Blue Book checks and the immunisations. It should almost be part of that checklist that mothers…

Female: Yeah that four year check.

[Over speaking]

Female: Doesn't it say has your child seen a dentist…

Female: It asks the question but it doesn't actually do anything to see a dentist. The thing is if adult - my beef a little bit is that not all adults can afford to go and see a dentist. It is very expensive to go and see a dentist if you have a limited income. If a parent doesn't see a dentist even for normal preventative cleaning and checks, then there's no way they're going to take their children along. I personally would love to see dental checks and preventative checks be part of the Medicare program and be bulk billed. Because if a parent goes regularly to

have their teeth descaled and cleaned and checked, then they will take their children. It will become as regular as it is with vaccinations and doctors' checks.

Facilitator 1: Do you think that there is an assumption because the parents can't access the dentist, that children don't have free access? Like there is a lack of awareness?

Female: Yes.

Facilitator 1: So they're assuming…

Female: Yeah.

Facilitator 1: So again the potential role of highlighting the study this is free for the kids is important, right.

Female: But that's not going to a dentist say in *** or whatever, they have to come to one of these centres?

Facilitator 1: No, they can just have follow-up anywhere.

Facilitator 2: [Unclear].

Female: [Unclear] but not a private dentist say on a main street, yeah.

Facilitator 1: No.

Female: I wonder if - on that note - whether it might be worth advertising through schools. Schools give news bulletins - whether advertising your program through the schools with the phone number and you might get a higher uptake of your service as well.

Female: Yeah.

Facilitator 2: The ECOH program is under five.

Female: Yeah for the public oral…

Facilitator 2: Yes.

Female: Sorry then the whole public oral health service program, that it's free for all school age children up to 18 I think it is?

Female: Yeah.

Female: That that be advertised too. But sorry the ECOH is up to five.

Facilitator 1: So with regard to other barriers that actually could potentially deter you from actually implementing the ECOH program. So you said probably the feedback is one thing, what about the actual process, the Lift the Lip, the documentation, the faxing? Are you comfortable - that's not a potential barrier to actually implementing the program?

Female: No.

Female: We're looking at an excellent booklet that has all the forms in it. But as someone else said, our biggest barrier is that we only see the motivated ones. I guess the ones that we really want to target - and I don't mean the really low because we capture more of the really low. It's the ones that just skim above the surface that don't come to clinic and we - so you know the preschool may be a place where more routine checks could be had for pre-schoolers. Because at least they'd get one check-up at preschool whereas we only see them probably till they're about three and that's if they've got a new baby. Otherwise we might only see them until they're two.

So really parents try hard for the first couple of years to be - good nourishment for them but once they hit preschool and start on the cakes and lollies the parents think oh well you know I've done what I can. So it's that preschool four year old, if they had a routine four year old check at preschool that would be a - you know really a good thing.

Female: I was in the era where I was checked at school, the dentist came.

Female: Yeah that's big school though, I'm talking about four year old.

Female: That was big school, I know it was big school.

Female: I mean even advertising on Centrelink payments or shopper dockets, there can be other ways that people are accessing information so that they know that the program is out there. I'm not sure what's on the back of the Lift the Lip brochure. Is there a referral number? If the parents find [that there's a problem] on the Lift the Lip brochure so that they could call.

Female: [Unclear] medical centres and doctors and maybe getting the word out through doctors' surgeries and other places where they do attend.

Female: Also we've lost the identification because I don't know how long ago it was the *** Dental Centre closed in *** . People still ask me about appointments for that place and it's closed how many years ago now?

Female: I don't know but I get asked the same.

Female: I still get asked about that and they…

Female: Was it a dentist's service?

Female: Yeah [unclear].

Female: They'd just go up there, walk up the path and go and talk to the girls. No drama. Now I don't how many years - they still ask if it's up there and they can go up there. People went there from miles around to be at that place. They decided oh no, too hard, we'll just close it down. Then programs like ECOH have come on but we still haven't replaced that branded recognition that that's where you go when you've got a child under five and that's where you go when you've got a child over five.

I think we're all sort of getting close to the same issue, that's identification of the service and accessibility to that. But I think they did used to know you go up to *** , just walk in and they'll sort it out. You'd have no teeth in your head or a toothache and they'll sort you out. Whereas we've lost that when we lost those services in [unclear].

Facilitator 1: What about - you know [unclear] it came about that we all have workload issues? Do you think it is just an added thing to your already busy…

Female: No it makes it easier. I think it's much easier, it reminds us to check. I think it's great.

Female: No trouble.

Female: Initially when we were given the forms to fill out we had drop-in clinics and we were seeing sometimes up to 20 people in one clinic. It was

very - it was tedious thinking I have to fill out this form and fax it off. But now we have appointment clinics we don't see as many clients, which is another story. But I find it easier now and I'm happy to do it now. But I will admit that initially when the program started I thought how tedious filling in another form and faxing it off. I'd rather just give the parents a phone number - and that's what I initially thought. I don't do that anymore.

Facilitator 1: What about training requirements? Are you- do you feel you're adequately trained to undertake this Lift the Lip process or do you think that's a potential barrier?

Female: No, there's no training involved. You just lift the lip and have a look at it. There's no training involved.

Female: It says on the form too, if you're not sure, just to refer. So it's not a drama.

Facilitator 1: So that's not an issue?

Female: No.

Facilitator 2: Do you have parents asking questions about oral health when you do the Lift the Lip or do you think that you have adequate knowledge to give them that information? Do you feel that maybe you want more information to give them information to some questions that are asked constantly that you don't have answer to, or anything like that?

Female: No.

Female: The main question - well everything is basically - like we're trained nurses so we have been trained in nutrition and oral health and all that sort of thing. But the main things that they ask me all the time is do I have to give fluoride? Because we have a lot of country properties that are not on the main water supply and that has changed. When I was first doing this we had to bring it in I think when they were two or something. Then they put it back to five, so that's the only thing that changes a little bit, is the fluoride recommendation.

But other than that I think we're pretty well equipped to answer any questions.

Female: You can also the - oh what's it called - the circular with the latest fluoride, that's what I do. There's a New South Wales Health circular on fluoride recommendations so [unclear].

Female: But now it's just - they use that on toothpaste don't they instead of…

Female: Yeah.

Female: There's no more fluoride drops or tablets or anything.

Female: But that's the only big change really isn't it?

Female: The most common question I think I get asked is when do I use toothpaste.

Facilitator 2: What do you say to that? Do you have that…

Female: Eighteen months.

Facilitator 2: Is that standard, everybody has the same…

Female: Yep, 18 months [unclear].

Female: It's on the Lift the Lip [unclear].

Facilitator 1: So apart from getting feedback from the public dental service, is there any other assistance do you think that they could provide to support you in implementing the ECHO program? Are you happy with the wait - there is no wait but you know for them to access the services?

Female: We could have toothbrushes to give out.

Female: Yeah it would be really nice to have toothbrushes and little things of toothpaste.

Female: We used to have chopper teeth. *** and I would talk about chopper teeth. I don't know if anyone ever stole anyone's or still got some. Gee they were good, big plastic things and you had the great big toothbrushes.

Female: Charlie choppers.

Female:	Charlie choppers and they were the best things because the kids were busting to go to the chemist to buy the little one. If we were lucky we had little ones in the drawer too. But that was really good.
Facilitator 1:	Something to instil interest…
Female:	You know what I'm talking about. [Unclear] it was a chore.
Facilitator 2:	They're still there, we still use it.
Female:	Well we don't. We used to have one of them in the clinics and they were like [unclear].
Facilitator 2:	[Unclear] having dental products in with you would promote oral health?
Female:	Yeah.
Female:	[Unclear].
Female:	We can't give them lollies if they're good so…
Female:	We give them a toothbrush, that's…
Facilitator 1:	The current resources you feel are adequate in terms of brochures and you're satisfied, you don't think…
Female:	Yes.
Facilitator 1:	So you've all received some sort of training with the ECOH through presentation. Do you think that is adequate or would you feel that you need more frequent training of this? Or would you want some sort of resource book or something like this you can carry around if you need to clarify anything or…
Female:	We've got the resource book really. I mean it was given five years ago I think. But there's still a few around.
Female:	Lift the Lip, the brochure itself is a good resource. It's got the information there, it's got photographs to refer to. I'm not sure that I would use anything other than the Lift the Lip brochure anyway. I think it's very self-explanatory.

Female: The other thing too is you can explain that this is the sort of teeth you want to have, not teeth like this. So that's what I like about Lift the Lip, because it shows you a really nice set of teeth and then those horrible set underneath. If you don't it this is what you're going to end up like. Some parents really listen to that.

Female: You could put some worse photos on there. Sometimes the photos - I don't think the photos on Lift the Lip are as bad as what you see out there sometimes. I think you could put some really dreadful ones on and I think it might motivate the parents a bit more.

Female: Like for smokers. Shock them.

Facilitator 1: So currently the ECOH training is done when the need arises. Are you happy with that process or would you prefer a regular - I don't know, once every three months or four months of someone coming down and giving you a refresher course? No, are you happy with how it is now?

Female: That's too often.

Female: We do have our nurse orientation three times a year so that new staff have that training and that is also opened up to any staff that are currently employed that just want a refresher. So not only with the Lift the Lip but with the steps, with the vision or the hips, you know we offer it to everyone who wants it. It's very comprehensive, the girls come out and give the program so it's always there. But as I said I think it's stuff that we do every day and we're pretty au fait with it.

Facilitator 1: Is there anything else?

Facilitator 2: Do you think you could - something would help you refer more often or you be able to refer more people or do you think somebody gets missed? Or are there any reasons why you might miss somebody? I'm just trying to see if there are any barriers that have come up that we can help.

Female: Yeah they don't come to the clinic for us to see. If we see them you'll see them. But if they don't come you can't do anything about that because it's voluntary.

Female: We see a lot of babies under 12 months and after that people don't come very regularly, if at all, and especially - if it's their first child they might come regularly. But if they've got other children they might think I already know all this stuff, I don't need to come to the clinic anymore. So they just don't come to the clinic.

Female: They'll do the three-year check when the little one is like one or 18 months because they're already taking the baby. So they'll bring the older kid along for the ride.

Female: That's when I do the opportunistic [unclear] when the siblings come in.

Female: I think there was some confusion with giving them out the phone number as compared to the difference with the faxing off the actual form of referral.

Facilitator 1: Was that a recent thing because I was just wondering whether that's reflected in the fact that the referrals have increased in the last quarter?

Female: Yeah probably.

Facilitator 1: Okay, so the confusion over that.

Female: I think it's really important to remember what everybody is saying, that the ones we really need to see the most - we see the bottom sort of 10 per cent of the population that we all need to look after. We see the ones that will come to clinic. But the ones that are really important are the ones above that bottom 10 per cent. They're the ones we need to find.

Female: So how do we do that?

Female: They all go to medical centres.

[Over speaking]

Female: …to see a doctor, I think the doctors need to be pushing this system.

Female: Sorry?

Female: I think the GPs and the medical centres should be pushing this. They all turn up to the doctors at some point. They may not come and see us but they will turn to a doctor when they're not well. If the doctors are aware that this service is out there and all children are able to use this service, then they may refer it more frequently as well.

Female: Also there are nurses at chemists who do the Blue Book checks so I don't know if you have access to those nurses and targeting them as well.

Facilitator 1: So using other avenues to [unclear].

Female: Obtain referrals.

Female: Yeah and emergency wards, you know there are a lot of children, you know febrile and children going to emergency wards after hours.

Female: Say ah.

Female: Yeah say ah, well that's it, they look at their throats but do they look at their teeth? I don't think they do sometimes.

Female: There's a nurse called *** who's at the mall in *** at the chemist in there. I'm sure - and others agree with me - but I'm sure that people who are more vulnerable will go to *** because they know she's not Department of Health. So if they can just lob into the chemist, talk to *** , she's very warm, she used to work with us, they all love her. I've never heard a bad word about *** . But people like *** are the ones - and she does one at *** too.

Female: She does one here at ***.

Female: She does *** down there. So people like *** - because she's not involved in the Department - those people who know the system will flock for their third and fourth babies to *** . Whereas we haven't seen them for years. Then you'll come across the oh no, I've been seen [unclear] see her … before. But I think they're the ones that *** says that we're going to…

Female: So why are they seeking her out rather than someone from public health?

Female: We ask too many questions.

Female: She's not [unclear].

Female: We're mandatory reporters and they know it. So you ask if there's guns in the house before [unclear]. They know that we're part of the great big institution that just groans along. *** is a free agent, employed by the chemist, come in, do her stuff, see you later … She is a mandatory reporter too but they're not as aware of it.

Female: It becomes part of the local knowledge.

Female: Just go and see *** down the chemist.

Female: I think the preschools because especially anyone that's had - people are putting their children in daycare under one year of age now, just so they can have a day off. So these children are going to daycare a lot younger than they did years ago so I think daycares would be a really good thing.

Facilitator 2: There's a separate program for daycares.

Female: Oh good.

Facilitator 2: Yeah, we call it Little Smiles and it started in early childhood centres. So there's different programs for different age groups in different centres - again for primary school, then again for secondary school…

Female: Although I guess it's hard because the parents aren't there, so it's hard to educate the parents…

Facilitator 2: Yeah, it's training the childcare workers and then so they do the Lift the Lip for early identification and then they inform the parents. There's a referral form for the parents and that in their book as well. So the pathway for that is [unclear]. But ECOH means the under-fives seen by child and family - I take your point that doctors and other people whom these parents go to should also be involved in it, including [unclear].

Female: Yeah referrals would be greater I think if you targeted the people that they visit more regularly.

Facilitator 2: The doctors, yes.

Female: The car seat checking stations. Everyone has to get a car seat check now so maybe we can do a dental check at the same time.

Facilitator 1: You've covered very important points, so is there anything else you'd like to - any other issues we may not have touched on that can…

Female: They don't like hearing soup - that they'll eat soup by the time they're 20 if they don't look after their teeth. That works, they come back and say oh I remember you saying that. If you can't chew meat you've got to live on soup. That's really effective.

Female: I do think there's a perception out there that any mouth - any tooth problem can be solved. You know it's not about preventative - I don't want to have to replace my teeth with dental implants or have my teeth whitened because I stained them or - you know with decay. There is a perception out there that any problem can be fixed with your teeth. They don't realise that it comes with a great deal of pain and expense to keep your healthy teeth there once they've decayed out of your mouth. It's very expensive.

Facilitator 1: So the preventative mentality is not there you mean…

Female: It's not there, I don't think it is there as much.

Female: Link it to a Centrelink incentive and you'll get [unclear].

Female: That's what I reckon too. Because they're the targeted people.

Female: I think a free toothbrush will bring them in.

Female: You were asking about a certain percentage of people that we're not seeing and we have actually made the service very difficult to get hold of. Because they can't - they just can't afford to spend a lot of time on the phone, they can't afford to ring and make an appointment because it takes so long to get onto the intake. So there's a whole section of people that we're actually not seeing at all because they just don't

have the ability to ring up and spend hours on the phone waiting to make an appointment.

Female: That's naturally - you're going to get a natural drop off of referrals because we're only seeing the people who are taking care of their teeth and just want their Blue Books filled out.

Female: We've got lots of [unclear].

Female: Just the well babies we're seeing.

Facilitator 1: Well thank you so much for your comments. Have you guys all filled out the demographic sheets…

Female: Yeah.

Female: Can I just ask one question? You can only use that form if there's an actual problem, you can't refer them for preventative can you? Like if there's nothing…

Facilitator 2: You can. If you think that there's a high risk child and that the family is - I mean if the family is going to private dentists or has their own and there's no problem then we don't want to clog the system. But if you think that the child's family is high risk and the child - you cannot [unclear] now but is definitely someone who will get something down the track, by all means yes. If … possible … then once a dental therapist … teeth. The therapists I have to say are very motivated. They're the ones who'll see the children, dentists don't see young children. They're all seen by therapists and they are - like they carry torches and do everything to get the child's attention [get parents'] attention.

So if we can get them in the chair, then take care yeah. So if you have a doubt by all means do. But please remember they're not [unclear] the people who are … this or wanting to see us. So we want to … people who will benefit and who will come and…

Female: Yeah [unclear I'm thinking but nothing has happened to the teeth yet but I'm looking at mum's and [unclear].

Facilitator 2: Yeah but if you think it's a high risk child, a high risk family, yes put high risk family and then just send it and that's a good reason. That [unclear].

Female: So the only one who has teeth in the family is the baby.

Female: There was some confusion about whether the parents needed to sign the form and I've never bothered with that.

Female: No.

Female: But they don't do that, no.

Facilitator 2: It's for you to refer to us, that's…

Female: As long as they know about they'll get a phone call and…

Facilitator 2: Yes, [unclear].

Facilitator 1: Thank you so much.

[Aside discussion]
