# Supplementary material for: The early childhood oral health program: a qualitative study of the perceptions of child and family health nurses in South Western Sydney, Australia
Source: BMC Oral Health. 2016 May 16;16:56. doi: 10.1186/s12903-016-0213-0 (PMC4867529; doi:10.1186/s12903-016-0213-0)
Supplement: Additional file 2: — Transcript 2. (PDF 282 kb) [file 12903_2016_213_MOESM2_ESM.pdf]

Transcript 2

START OF TRANSCRIPT

Facilitator: Is there are red light?

Female: Yes.

[Aside discussion]

Female: So funny.

Facilitator: It says: if you feel uncomfortable or upset, the focus group will stop and [you may be able to be] comforted.

[Laughter]

Female: Come back and say: there, there.

Female: It's a [unclear].

[Laughter]

Female: Continue now.

Facilitator: A lot of these things are required by ethics and they have a standard set protocol, because sometimes focus groups have sensitive information that they discuss about how do you feel like being a carer for a person who has cancer and has died. Looking at the stress level of the carer and how that stress level then impacts the outcome of the patient. Because if they're surrounded by family that is constantly stressed, then the patient themselves, is even more stressed than them, they have worse outcomes. So sometimes the carer can just break down and feel really sad and so we have to stop, start. But we are discussing no such thing today.

Female: [We don't want any nonsense].

[Unclear] words are denoted in square brackets and time stamps may be used to indicate their location within the audio.

Distribution of this transcript requires client authority and is subject to the provisions of the National Privacy Principles.

[Laughter]

Facilitator: We're here to talk about kids and make sure that they have nice smiles for a long, long time and what you and we can do together.

So the information sheet is for you to keep but the consent form we collect it for our records. As I said: if you do not want to participate and you're not coerced into sitting here and you're happy to talk to us.

Female: [Unclear].

[Laughter]

Facilitator: Please don't say that, the mic is recording.

[Laughter]

[Over speaking]

Female: Sorry. Well it says on number three…

Facilitator: Yes.

Female: Relating to any possible physical or mental harm I might suffer. What would I suffer [laughs]?

Facilitator: Yeah.

Female: So what age group will that focus on?

Facilitator: Which one?

Female: What age group? For now.

Facilitator: [Unclear]. We are talking about ECOH so [unclear] for results for children under five, because children under five are not seen anywhere until they reach school and sometimes it's too late. They are either into bad habits, into bad dietary and their teeth are already starting to rot. So this is the only way we can get children early and childhood family nurses see many children from birth and as often as - I mean that you guys are one of the first, at least point of call to alert us, saying hey something is not right. So yes, this is mainly for children under five. Over five they have school programs and check-ups and stuff so they get covered under that.

Now I will start officially. My name is *** and I'm from *** . We used to be the same service but now we're splitting, so I'm based in *** Hospital at this point but I still oversee all the projects that we are already involved in for both Sydney and South West Sydney [LHD] and this is ***.

Facilitator 2: I'm ***. I'm from *** and I'm a lecturer in *** there.

Facilitator: We are a small group so quite informal. So feel free to introduce yourself.

Female: I'm *** I'm a childhood nurse.

Female: I'm *** Same as *** childhood family health nurse.

Female: My name is *** I'm a childhood [family health nurse].

Female: I'm [unclear]. We've already met…

Facilitator: Yes.

Female: …I'm a [unclear].

Facilitator: Okay so what I thought we'd do or we thought we'd do is - have you had ECOH training, heard about ECOH, done any [courses]? Is ECOH a new word for any one of you?

Female: [Unclear].

Facilitator: It's Early Childhood Oral Health, that's what it stands for. It's been implanted since 2007 and the main aim as we discussed was to reduce early childhood caries using childhood family nurses as well as GPs as our point of contact, first contact. What the aim is basically, to reduce the incidence and prevalence of caries in young children, especially high risk children. What has been put in place is that any child family nurse who joins, we try to give training once a year, so somebody comes out, gives a talk what early childhood oral health is about, what are the key things that a nurse should look for. Like a simple thing as Lift the Lip. Just see.

Because the reason we do Lift the Lip is if you see cavities in the front teeth of a child, that means it's a really severe case. Most of the

cavities happen in the back because of the chewing surfaces in the back. So the sticky food sticks at the back so some children do get cavities if their oral health is not the best. However if the practices are not right like child is feeding from a bottle, sugary drinks all the time so the sugar is there in the mouth - all parts of the mouth, not just the back then we start seeing cavities. Now a child's tooth is much smaller. The cavities progress much faster and the sad part is they cannot express themselves.

So if they're having pain they just won't eat anything or they just turn their face away or are grumpy but they cannot say pain, say my tooth is painful. My lower back molar is painful for example. So you guys when you're doing a check, you can just quickly lift the mouth and if you can see an obvious black cavity that's raising alarm bells that there's something seriously wrong in this child so best to refer them to an oral health specialist to look after them. If you can take a bit more time and if you can spot white lesions, that's even better because white lesions are the first step of cavity.

So if a child has a lot of white lesions we can still reverse the process if they can be seen by us quickly. By the time they become black it's gone. The tooth is already decayed but if it's white, it's still intact. We can reverse the process so the child can still have his teeth until they have the permanent teeth come through. It's also about educating the parents that yes the child teeth will fall and a new one will come but if the child's small teeth are rotten, even the adult teeth will rot, because the mouth doesn't change. The teeth might change but the [unclear] remains the same. So it's about giving them that information and if you can start the referral process then we take over and we give the child regular maintenance and [unclear].

So that's what ECOH does. It's basically working in collaboration and seeing if we can identify. So you guys are the main people because you see them and you can identify. If you can identify and refer them, we can get the ball rolling. Okay so as I said ECOH program has been here since 2007 and we have referrals and it's steadily

increased. Every year we go out to all the community health centres. If they have new staff then they have courses and we've got [a few] new staff and we can give them training. So almost all staff are trained about ECOH. It's in the Blue Book to ask at the same time, if you can just ask.

If you don't want to lift the lip, the mother can lift the lip or you can use a gauze to lift the lip, whatever you feel comfortable and the child can help you too. There's positions so sometimes the mother can hold the lap and do it or mother sits on the side and you on the other side and you have a look. So those are very basic requirements of - in doing the oral health exam for a child.

Today we are here because we want to ask your opinion as to how you think ECOH is going. Is there some place for improvement? Is there some barriers that you feel…

[Interruption]

Facilitator: So if there are barriers that you have or anything that you would like to give us some ideas so we can take back and see if we can improve this communication that we have and this relationship we have. So that's the whole point.

[Interruption]

Facilitator 2: So what we're going to do is - this is a focus group. I guess we might go through some of the rules of the focus group but does anyone to leave? I should say that before we go any further? You're all happy to stay? Okay so we're going to try and run it in an hour. We'll run about an hour. It depends how long you talk and how long the boss lets you have to do it. So just the basic rules - when we're having a conversation if people don't speak over each other. It's hard to record the ideas. What else? Don't use people's names. So when you're referring to each other or people that you meet in your practice, if you could try not to use their names.

We'll send these recordings away to be transcribed and we'll remove all names anyway. There'll be no way of - you remain anonymous in

the program too. So you don't have to worry about anything that you say that you think is a bit sensitive and it being tracked back to you, okay. We're going to get you to give us a bit of demographic information as well. So that will be your age and I guess you don't have to say your age. I think it's a legal thing that you don't have to give your age but if you're happy to - or in a range, that would be good. How many years you've been a child and family health nurse and what your highest qualification is.

So everyone here works just at *** or do some of you work at other places?

Female: This is the main base but they do go to outreach clinics.

Facilitator 2: Outreach clinics okay and full or part time?

Female: Full time.

Female: Full time.

Facilitator 2: Full time okay and you've all had ECOH training.

Female: Yep.

Facilitator 2: Okay, what happens when new staff come in? Do they have to wait until the training runs…

Female: Yeah.

Facilitator 2: So they might have a whole year before they can do it.

Female: Yeah.

Facilitator 2: Do you think that perhaps we could be looking at that differently? What would you say? What would work best for you as a group if a new staff member came in?

Female: Unfortunately we don't get many new ones and it would only probably be one.

Facilitator 2: Right. So in that situation would you orientate them then too?

Female: Yeah they just go through our normal orientation and they'd find out about it.

Facilitator 2: Okay, oh good. Okay, well thank you for participating I should say first off. Now we've got a set of questions that I'll ask but I'd really like this to be a talkfest so jump in where you feel you need to jump in and elaborate where you think something needs to be expanded on. So we might just get going if that's okay. We're about to begin.

Facilitator: Yep.

Facilitator 2: Okay.

Facilitator: As we said, all the information that you provide is anonymous. When we transcribe your comments we just put the comments. It doesn't say who stated it, which clinic it came from. It's completely de-identified, so if you would like to criticise, say this thing can be changed. I'm not happy with this, feel free to say it, because that is what we are interested in. We are interested in [terms] of your thoughts so there is nothing that will be published or discussed with your names on it. Only your ideas will be discussed. Okay, alright.

Facilitator 2: Okay so we've got the consent forms.

Facilitator: Yep, we'll collect it. If you could just pass it to ***

Facilitator 2: Have you had a chance to finish your…

Facilitator: Otherwise we can do it. I will be collecting as we discuss.

Facilitator 2: I grabbed a couple of extra pens out of my bag. Here you go and you're not going to do it are you?

Female: Huh.

Facilitator 2: You can't [grab it]? Oh you have, alright.

Female: And the demographic information?

Facilitator: Yep, do you have that? Do you have a copy?

Facilitator 2: Just send that around to fill in as we go.

Female: Do you want us to swap over from signatures or witnesses or…

Facilitator: That's okay. No we can. One of us will sign it. So you are here.

[Aside discussion]

Facilitator 2: We might need it for the demographic information. Okay so we'll just get started. So our first question is…

Facilitator: Did we go through what their requirements are - I mean the [unclear]?

Facilitator 2: So actually the aim, the official aim is to explore the views of child and family health nurses towards implementing the ECOH program in South Western Sydney. So I went through the fact that it's going to be taped. That it's confidential, that it will be transcribed. People will be de-identified so we'll just go into the…

Facilitator: Yep. So it's all…

Facilitator 2: All set yeah and they've been given a chance to leave if they want.

Facilitator: Yep.

Facilitator 2: Okay, alright so the first question is in your opinion or experience, what are the benefits of good oral health for infants?

Female: There's a lot.

Facilitator 2: There's a lot.

Female: They need obviously their teeth as they get older for eating. So they need good oral health for that. Also they can get infections through bad oral health and…

Facilitator 2: So what sort of infections are you talking about?

Female: Infected gums.

Female: Yeah, infection.

Female: Swollen face from the teeth.

Female: Obviously pain, so being unsettled and things that could lead to the unsettled behaviour and long-time adult oral health problems. Yeah that's the main ones. Obviously if they do have bad oral health, they have to get treated and that's quite painful and both parents and the child - so going to these - getting oral health care which they could obviously avoid it all together.

Facilitator 2: Does it have an impact on them when they go to school? In what ways would that have an impact?

Female: On bullying I guess because they're mouths would look different to other children and makes them a bit more introverted because they're reluctant to smile and make friends, talk, communicate.

Facilitator: Appearance, for eating, for prevention of infections. Why else do you think teeth are important?

Female: Oh yeah speech. Speech, as well talking.

Facilitator 2: Okay so in your opinion it's an important consideration from early on, yes?

Female: Yes.

Facilitator: Anyone game to say no. Okay so what do you think is the understanding of parents? So if you see a lot of people from different backgrounds, do you think that generally speaking, parents are aware of the importance of oral health or does it really vary?

Female: They are aware. When they come to the clinics, we do mention to them about their teeth and we'll ask them to read their Blue Books because it's all in the Blue Book. When their teeth comes, what you need to do. Also we do advise or discuss with them how to care for their teeth.

Facilitator: Okay.

Female: If there are any problems they ask us.

Female: I think because they think it's their first teeth too, then it's not going to affect them later, it really doesn't matter.

Facilitator 2: Okay so is that a common misconception is it?

Female: Ahem.

Female: Some don't actually know when to start cleaning their teeth. They can think okay it's too young but we tell - I tell them that as soon as the teeth come out, you should start introducing cleaning their teeth for them and…

Facilitator 2: Can you give some examples of what parents have said or what they say when they…

Female: Is it too early? Is it too early to clean their teeth?

Facilitator: Do you remember any conversation with the parents? Please feel free to share to give us an insight into what they think.

Female: Nothing comes to mind.

Facilitator 2: Okay so do you think that child family health nurses have a role to play in promoting infant oral health and what do you think your role could be in this area or is?

Facilitator: First do you think you have a role to play? Do you think there will be any added benefit for you to look into the mouth because you have so many things you have to see and sometimes the child is very cranky? You're talking about young children. Not all the time these young children can sit in the chair and open their mouth or whatever. They are squirming and they don't want to be in the room and stuff like that and you have to do all those checks so do you think you should spend a few minutes to think about oral health? Do you see that it's worthwhile, that you can make a - or no? Or you think oh well, it just really doesn't matter because…

Female: We always discuss that in the Blue Book. I mean it's [a format] in the Blue Book anyway and there's always a discussion of who cleans the teeth or sometimes it can be they feel the child hasn't got a full mouth of teeth so there's no real reason to clean it. But as *** said, you know as soon as you've got one tooth in the mouth then you start telling them to clean it. But yeah it depends on the parent really as well.

Facilitator: But do you feel that by asking that you are going to make a difference? Have you seen a case where you have noticed something and done a referral and it's turned out to be good or most of the

children who come here have relatively good teeth and there's not that much of a need.

Female: I've probably found parents have been wanting to give - one couple has come into mind at the moment, where the child wouldn't eat anything but chocolate. So if the child is only eating chocolate, where is it coming from and who offered it in the first place?

Facilitator: That's right.

Female: Apparently the father had introduced the child to chocolate and didn't see any problem with that - an under one year old. So we just got the chart out and showed him and what could happen and went into the cost, the possible cost to his child. But he really didn't see any issue with it.

Facilitator: So did you expand as to what might happen?

Female: Oh yeah. That's why I said. I got a picture out, showed him the rotting teeth…

Facilitator: Teeth - but that the child can end up in a hospital with a swollen...

Female: Yeah but it was a baby. It was a young child.

Facilitator: It doesn't matter.

Female: He didn't feel that there was any issue with giving a child his chocolate.

Facilitator 2: So it was going to continue you thought?

Female: I actually don't think it would after they left because.

Facilitator: Oh good.

[Laughter]

Facilitator: Because not the just teeth. Anything bad for your teeth is bad for your health so from a nutrition point you must stress the same thing as well.

Facilitator 2: So you haven't seen that baby in a follow up yet?

Female: No and sometimes they don't come back. We tell them off.

[Laughter]

Facilitator: True.

Facilitator 2: Okay so you think you all have a role to play. It's an important role to play. Do you think there could be anything else you could be doing as part of that role?

Female: I think we could introduce - you know the face washer and tooth care and stuff like that with solids and stuff don't we really.

Female: Yeah.

Female: Play and as the child grows and grows, you put them up on the sink and get them to mimic what mummy or daddy is doing. I'd agree with the girls. The Blue Books - I think because we probably as a group, start very early with that introduction to tooth decay and tooth problems and speech problems that we tend to be picking up maybe a little bit earlier and preventing probably more so than anything else. Don't you agree?

Female: Yeah.

Female: Do you remember when we had that survey a few years ago where we were offering the parents a 12 month check and a free toothbrush and whatever? It was quite interesting to see the amount of parents who didn't want to sign up for it. There was quite a few who did but when you really think about it, they were the people who were the regulars anyway. So they're the ones who really cared. Whereas the ones who were refusing were the ones who would think oh yeah, he's 20 months old now. Oh I didn't do the 18 - oh I didn't do the 12 month check either. Then they suddenly appear at the clinic. So they don't really follow through with every Blue Book check.

Whereas the ones with - going back to the survey, the ones that seemed to be really eager to take it, were the ones who are going to come every time for a Blue Book check.

Facilitator: Yeah so mainly people who take advice are ones who are anyway quite - I mean is interested in making sure that the child's got good general health and good dental health.

Female: Not particularly more educated but more wanting to be educated for their children's sake.

Female: I picked up a lot of older children like the babies come in but I've actually dealt with the siblings and that - it amazes me how they've gone through without being picked up early. I've had good positive response, [being checked] by a service and getting treated quite quickly.

Facilitator: That's good.

Facilitator 2: Do you pick up too on the parent's oral health while you're having those visits?

Female: Yeah.

Female: Yeah we do with drug and alcohol.

Female: Yeah and because they've had such a negative experience that their perception that their child's going to have that negative experience as well with the dental system. One lady who comes to mind is quite an articulate, well-educated girl or woman that had been trying for 12 months to get something done about her teeth. I think she gave up in the end.

Facilitator 2: So…

Female: Because of bad appointments or she didn't make it or family interventions and the whole scenario of problems so yeah. I think trying to educate that lady about the importance of adequate dental care would be three times more difficult than trying as *** said. Women that actually come to clinic - I don't think they're the ones that you need to be targeting. It's the ones that are hidden. It's almost like an occult.

Facilitator 2: So why do you think people that might not be coming? So you're seeing people who are already convinced and you know are going to

be proactive. So is there a big group out there that you're not seeing do you think?

Female: Well I think and I'm not saying all but I would say that a portion of - a clientele that I see.

Facilitator: So when do you see the big fall? So at six months you would see a large number…

Female: I'm talking about parents.

Facilitator: Oh parents okay.

Female: Because to me I think what happens with parents impacts on the child.

Facilitator: Yes.

Female: Does that make sense?

Facilitator: Yes.

Female: Mum's are going back to work earlier now as well. So someone who might follow through for the first year, then they'll go back to work and we don't see them again until the four year check so you've got a really big gap. Grandma might bring the child but more often than not, they've gone to child care and so they…

Facilitator: So after 12 months there's a big drop.

Female: It can be sooner than that. You can get mum's going back to work after three months and then you just don't see the children anymore.

Facilitator: Is it the same for the first child or is it more often for the second or third or it doesn't make…

Female: I think it's the same with all of them.

Facilitator: Okay.

Female: I saw a first time mum last week who was going back to work at three months. So it's not uncommon.

Facilitator: So the Blue Book remains untouched?

Female: They use their GP more often than not because then they go for the immunisation, the doctor listens to the baby's heart and gives them a once over. But we find quite often they might go to the GP for their check but then they'll come back to us provided they're not working for a re- check because they didn't feel the GP did that or the GP didn't do that so they like to have it double checked.

Facilitator: I did the same. GP didn't do anything. I always take - I get more information with the [unclear] who can sit down and tell me. Yeah okay, but it's not about me.

Facilitator 2: So do you get doctors referring these people back to you?

Female: Sometimes we do.

Facilitator 2: Yeah for their checks.

Female: Yeah.

Facilitator 2: Or do the doctors do checks as well?

Female: They do both but sometimes they'll tell them to come back to the child family health nurse because especially if the baby is unsettled, rather than them write a referral, they'll send them back to us for us to write a referral. Yeah that's probably the main reason that they would.

Facilitator 2: Okay so people aren't coming to see you. Where are they going do you think? Do they go anywhere else to get advice about…

Female: That's what I was saying, the GP.

Facilitator: GPs yeah. Anyone else?

Female: Chemist.

Facilitator: Can you tell me about that?

Female: No because I've never been to one.

[Laughter]

Female: It's just that they've got a - they employed a nurse at the chemist. We don't know what training they've had. They won't have had lift the lip

or anything I wouldn't imagine. Unless they are a child and family health nurse, it's just an extra job - that would be the only thing.

Facilitator 2: So why would they go to the chemist I wonder?

Female: Convenient.

Facilitator 2: Convenient. Why would they go?

Female: Drop into the shopping centre and just the baby weighed and…

Female: Yeah.

Facilitator: Okay so it's…

Female: If they're in the shops anyway, just go in. Shove it on the scales, there you go, more or less.

Facilitator: Okay.

Female: Sometimes…

Female: There's no consulting room anything. You're just out in the shop.

[Over speaking]

Facilitator 2: Public area.

Female: Yeah.

Facilitator 2: So do you think that people are going to - the standard dental services, rather than come into the clinics? Do you know, like going to their family dentist instead when they have [inaudible]?

Female: It's possible.

Facilitator 2: You haven't heard anyone talk about that?

Female: Not really heard about it, no.

Facilitator: But do you ask have you taken your child the dentist? Is it part of anywhere in your checks?

Female: Not really.

Female: No. I mean if you get a child who's got virtually no teeth at [15] months, then you're going to start advising them to maybe go and see the dentist. But not really to go and get a health check as such.

Female: We hand out the [leaflet] as well and they're quite specific. If you see the white bit, get up to the dentist and if you see the brown bit, you're in trouble.

Facilitator: Correct.

Female: So you hand those out.

Facilitator: Okay.

Female: Most dentists are expensive too for them so if it was free, it might be different.

Female: Yeah. We do have brochures for services for under 12 year olds which I think you give them out at your solids group too, don't you. I always give those out at the solids groups.

Facilitator: So do you know - do they know that the oral checks or all the treatments for children up to the age of 18 is free if you are an Australian resident?

Female: Some of them do. They've got brochures.

Facilitator: Because if cost is expensive, then if they happen to go to public dental clinic then it's completely free. All they have to do is ring the number and give their Medicare card and that's it.

Female: But how long's the waiting list?

Facilitator: It's come down from [unclear].

Female: Has it? Because I have a friend who took her child about 18 months ago and that would have been as long as the list was then.

Facilitator: Yeah because I think since beginning of last year, the funding has come through to completely get rid of the waiting list and the waiting list has come down for children dramatically. I don't think there's a waiting list in fact, anymore. So in fact it's good that it can encourage

them to seek help and it's free. It's about knowing - the knowledge. So if you want, we can send some brochures about free dental care.

Female: Yeah.

Female: I think another group of children too that probably slide under the radar are the children that are out of home care. They're the foster children. Sometimes they've gone from one home to the next to the next and they may never see a dentist until - if ever. So I think that they're a group of children too that are needy but maybe not seen as quite so needy.

Facilitator: So who brings them to you if they're…

Female: Foster carers.

Facilitator: Foster carers at the time okay. Do you think that there's a difference in their opinion and compared to…

Female: Depends on the foster carer. It's a very individual thing, in my experience anyway. What do you think *** ?

Female: So many foster parents are very, really good. Others it's a job.

Facilitator 2: I wonder what information they're given as foster parents in terms of their duty of care and…

Female: See their Blue Book's - sometimes those fosters carers aren't aware that six to eight week check or the - because baby's moved on shortly after that. So the six month check may not be done because they're just not familiar sometimes or they've got so much on their plate with the foster carer or with the child. Sometimes they've got two and three kids that are in foster care so it's worth thinking about.

Female: Is there an age that you recommend the kids at a certain age to get checked?

Facilitator: The NSW Health recommends that at age the of - by the time they're two, they should have their first check by a therapist. So all the professionals who look after children in public service are the therapists and they are trained to only care for children. So they're

really good and when a referral comes then they look at the whole family because it's part of the whole [strategy], it's not just the child. So they get into it. It's just a matter of getting their need. That's the main thing yeah.

Facilitator 2: Okay, so what exactly would you do in a typical visit and can you give us some examples?

Female: For the Blue Book check?

Facilitator: Say at 18 month Blue Book check, what would you be doing?

Female: Eyes, weight, height and then obviously the Lift the Lip. Talking about how babies eat, the child is eating, what they're eating because at that age they tend to get a lot of fussy eaters.

Female: Talking.

Facilitator: Sorry?

Female: Chatter.

Female: Yeah, whether they're talking or not which also can be related to what they've been eating. If they're still pureed food then highly likely that they have speech problems or if they're using dummies or if they're using bottles.

Facilitator: Okay.

Female: You try and encourage the mum's from six months to start introducing a cup for babies. Even just a drop of water, just to get them used to the idea and around about the 12 month check we start talking about getting rid of the bottle if possible. Also safety issues of carrying drink cups and bottles around once they start toddling. Because if they fall and they can knock out teeth. Eighteen months is the now let's try some toothpaste. Hopefully the child is up to spitting it out by now and also encouraging the child to start doing it themselves as well a little bit. Some of them it's still too early. Still too babyish really.

But you'll find that some mums will give the child the time to let them do a little bit themselves and they'll do it as well, or they'll have a fight.

Facilitator: So do they ask by when they should be alone, doing it alone or up to what time the parent should assist?

Female: Six or seven window of age.

Facilitator: Yeah that's right. Up to the age of eight they should be assisted and how you stand. You stand behind the child and do it so they don't just go running away [unclear]. That's good. So you talk about bottle and then do you go into nutrition? What's in the bottle, what do they drink.

[Over speaking]

Facilitator: Okay and what is yes and what is no? What do you recommend then?

Female: Well we don't really recommend juice - definitely not in bottles. No soft drinks in bottles. If they do have to have a bottle, if you try and wean them off them with water in the bottle rather than milk and you'll find as the child grows, most of them will still want to give that child the night time bottle. So then it's a case of, well really you should be cleaning the baby's teeth after the bottle.

Facilitator: Correct. Yes.

Female: That's what worries them a bit because they use that bottle to send the child off to asleep.

Facilitator: Any example that you have noticed, that's some interesting activities that they do?

Female: No.

Female: A lot of them give the toddler formula now too because they think that it's better for the child, so some of them are longer on a bottle than the cup. Really there seems to be a trend to give them the toddler.

Female: Yeah weaning off the bottle is hard. They use it for [comforting] - so to go to bed. That's why they're just prolonging it a bit longer. So getting them to stop is hard.

Facilitator: So do you recommend some things or you don't?

Female: No we just tell them we advise you not to and the reasons why. Just encourage them to - even if they gradually cut down the feeds from - like the daily feeds too. So big cups and then eventually cut it down totally.

Facilitator 2: So can you give an example of when one of your visits with someone went really well. What you did there or when it didn't go so well and it was difficult.

Facilitator: Not just dental, anything. What would be a challenging visit?

Female: When they start saying my parents did it or what's wrong? We've all done it and we're all fine. So just addressing this, it's really difficult when you try and argue your point.

Facilitator: That's a good point yeah.

Facilitator 2: So do you - when you do Lift the Lip, like you start do that at what time? At what age?

Female: We do it from the first, when they're about six months old you start to look at what's happening, whether they've got any teeth or not. But then on the initial assessment when you're doing your one to four, you're looking in the baby's mouth. You're looking at the palate, looking at the lips, looking underneath the tongue seeing if they've got any tongue tie. If there's any indication of thrush in the mouth, that kind of thing. So you do it on the first visit and some of these kids have their tongue tie snipped if they've got a tongue tie and you see them again and you might have a look at six weeks as well. So you really look at the tongue.

Facilitator 2: Do you train the parents up to do Lift the Lip or…

[Over speaking]

Female: They tend to do it anyway. They're always fishing to find a tooth. Some of these poor babies have this curled up lift.

[Laughter]

Facilitator 2: So for getting to the process for referral, from your experience. So what's the process you follow in terms of referring someone on?

Female: To be honest with you, I haven't really referred anybody for dental.

Facilitator: Okay.

Female: But I have advised people to see the dentist but there hasn't been anybody. There really hasn't been anybody for quite a long time I would say.

Facilitator: That's good. So you think most of the Lift the Lip checks you do not find anything really nasty? No. Okay.

Female: Maybe with some drug and alcohol ones. We get a few but not a lot. Maybe they should target the child care centres, preschools. That would be…

Female: Yeah that's a good one.

Female: It would actually. It's where you would catch them all really.

Female: The grandma's are looking after them and insisting on giving them an arrowroot biscuit every time they open their mouth.

Female: [Laughs] because it's not a real biscuit. It's only arrowroot.

[Laughter]

Facilitator 2: It doesn't have any sugar on it.

[Laughter]

Facilitator 2: I was brought up on arrowroot biscuits.

Facilitator: So you haven't recently referred anybody.

Female: Not for a while.

Female: No.

Facilitator: So if you have to refer, what would you do?

Female: I've made a few referrals actually but it's been a while. I usually just fill out the form and fax it. Then usually I get a letter saying, it would have all the client's follow up letter so that's great.

Facilitator: So do you find the feedback's useful?

Female: Yeah.

Facilitator: So you're glad to get feedback if you make a referral to see what happened to the child.

Female: Where do they have to go if they have an appointment with, or referral? Where's the closest dental health…

Facilitator: For *** it would be *** .

Female: *** okay.

Facilitator: Yep at the dental clinic there. *** , maybe *** . It depends where the child is, just convenient for them. There are clinics from *** all the way to *** , depending on where the referral is from.

Facilitator 2: In terms of that, are you aware of the eligibility criteria for that?

Female: No, which was going to be another question I asked.

Facilitator 2: Okay. So how would you find out what the criteria was okay.

Female: Yeah.

Facilitator: So what criteria are we talking about?

Facilitator 2: To be eligible to access those services.

Facilitator: Is this under the age of 18 and have a Medicare card. For the older people, they have to have a Health Care card. For children you don't need to have a Health Care card as long as they have a Medicare card.

Female: I think with the referral, when I was going through it last time - I think it has, if they've got a cavity or something that's got…

Female: Criteria.

Female: Criteria. So it doesn't really help for a normal general check-up. So that could be added, like another section saying just for a general check.

Facilitator: See you have to remember when you do an ECOH, you are catching child at risk of decay. So you have seen something happen and you want them to go and have a look. But if you want to play a role of referring children for check-up under ECOH, then I take that as a request that why not. When a child comes to you and you see that their diet is not - okay they don't have a cavity yet but everything else is ringing a bell in your held then…

Female: I'll just seek an opinion.

Facilitator: You should just [say] - and then you can just say check-up, okay I can make a note of that and see.

Female: Because that's one thing I was - I think last time recently that I was asking about - a Mum just asked for a referral and I was going through that. I was thinking whether this child would be still…

Facilitator: You can still do it. There's nothing [unclear]. They won't ring back and say you've not put a cavity so we won't see it, yeah but if they - you send a fax then that section only tells them how urgent the matter is. So if there is an obvious swelling then they have to get the child within 24 hours but if it is something that as you said, you're worried then they can ring them at their convenience in a week or two - when the child can come and the parent can come. So that gives them an idea but yeah I'll have a look at whether a general check-up is there on the form.

Female: I'd think you'd find that if the child was in pain, they'd go off to the GP first and then the GP would then refer them in.

Facilitator: Sometimes they don't. They just take a pill and forget about it until the swelling goes and that's the problem. Even for the adults, that's the…

Female: Then if we're talking swollen faces, I think you'd probably find that they would…

Facilitator: Go to the GP.

Female: Go to the health centre.

Facilitator: But then the GP should make a referral. Fill out the form and then send it because yeah, that's another part to it. We have explored going to the GPs as well, done the training for GPs as an online training for GPs as well. To give them continuing education points and also talk about ECOH.

Female: What about ante-natally? Because we were recently just picking up, me and *** were talking about mum's having gum infections and they get [unclear] bacteria that could be passed onto to their child later on. So a bit of education there would be helpful.

Facilitator: Because we're running a program in *** Hospital and now we expanded that to *** and what's the one in…

Facilitator 2: In ***.

Facilitator: Not ***. In west.

Female: ***.

Facilitator: No, no, beyond ***.

Female: ***.

Facilitator: Yeah *** Hospital. Where we're requesting the midwives to include oral health as one of the checks for the pregnant women and the first trimester. So come to something like Lift the Lip for them as well. So just ask them to open their mouth and show - there's two questions for them, asking if you have a problem or have you visited the dentist in the last 12 months. If the answer is no for the visiting dentist and yes for the problems, then there's a referral [out there]. The thing is right now public clinics do not have free dental care for pregnant women.

Female: You have to have a Health Care card.

Facilitator: If you have a Health Care card no problem, anyone can then do the referral and the pregnant woman can be seen in the public hospital. But if they don't, then they have to go privately and get checks done. So there's a trial happening first time in Australia here so hope to make findings will then result in recommendations that pregnant women should be eligible for free dental treatment.

Female: Because you said [unclear] went all the way around *** .

Facilitator: No.

Female: *** not included in that.

Facilitator: Not for the [multi] centre trial because it's just a research project that's happening now to gather information to support the recommendation.

Female: It just seemed odd that it goes all the way around and misses the big chunk in the middle.

Facilitator: It's just the hospital staff there are into some many trials, that they don't want to put their hand up for a new one.

Female: We've asked all the hospitals…

Female: Yeah, yeah it's interesting isn't it?

Facilitator: …to put their hand up…

[Over speaking]

Female: Because they have such a diverse culture and age group and requirement there for specialist care.

Facilitator: Yes.

Female: It's weird, isn't it?

Facilitator: But I think they have just so many trials, some hospitals they just…

Female: They're loss.

Facilitator: Over researched.

Female: Yeah that's right.

Female: The other thing I wanted just to clarify in my little brain. When you were talking just now about linking or answering *** question. Say if you had a seven year old child that came to you that had a really shocking mouth, full of cavities and whatever. If that child has younger siblings, you see them - you don't.

Facilitator: Absolutely.

Female: You do see them.

Facilitator: Yeah that's what the therapist does. If they find one child, then they will ask the mum to bring all the children and that's part of ECOH as well. So when they get a phone call from - you send a referral and then the dental hospital or the oral health centre then calls and says we'd like to see - we've got a referral to bring your child in. Do you have any other siblings or other children? Would you like to bring them for the check up as well and try to fit everybody?

Female: Good yeah.

Facilitator: Because it's not treating one child. If one child then definitely the whole family has something similar.

Female: You've got a reference point haven't you? Yeah exactly like that, it's sneaky.

[Laughter]

Facilitator 2: Opportunistic.

Female: Why not.

Facilitator 2: Okay so do you see - do you ask the parent the next time they come? You've given them advice, perhaps at six months, 18 months whatever. Do you check up with them that they've followed up or is that part of what you do?

Female: Usually. If we've noted something in the notes then we usually have a look at the notes before they arrive and just ask them.

Facilitator: So if you talked about bottle feeding and stuff and then you'll ask them at 18 months, what has happened?

Female: Yeah.

Facilitator: Okay and what do you get for a response normally? Does your…

Female: It depends on the mother really. Some of them are really good and they've done - one of the things that we try and impress on mums is to do, for the babies to do tummy time. The times that you'll have them come back and every time you ask them, they say doesn't like it. It really doesn't like it and you're doing the check and you can see that

there's a significant head lag there before you've even asked them. You're doing it and you say we discussed tummy time last time. Are you doing anything? Oh he really doesn't like it. Will do 30 seconds of it and that's it. So yeah, sometimes they do.

Some of them will go home and they'll really work at it and come back and before you even broach the subject, they'll say we've been doing lots of tummy time or we've been doing this. So they'll be ones like I said to you before - ones who want to be educated for their children.

Facilitator: Yeah and will come at every visit and bring the child.

Female: Yeah and they'll come between the Blue Book because it's to discuss things.

Facilitator: Oh right, so you can make an impact on the person who's receptive already but changing behaviour is a bit challenging or do you find some techniques are better? Do you have a technique that always works that you'd like to share?

Female: You can't force anybody to do anything with their baby. It is their baby in the long run so you can suggest and you can try and educate them. But in the end it's their responsibility, not ours for them to do what they have to do with their child.

Facilitator: Yep so do you give any reading material, any brochure or tools? Any other…

Female: Yeah we give lots of brochures out.

Female: The Blue Book's got a lot of information in there as well so they can read up a lot.

Female: It depends on the mum doesn't it?

Female: Yeah.

Facilitator 2: So do you give out things like toothpaste and…

Female: No.

Female: We're not funded for that [laughs].

Female: That did happen a few years ago but the dental hospital gave us a survey to do and offered the parents…

Facilitator 2: An incentive.

Female: A check and a toothbrush and a toothpaste.

Facilitator 2: Do you think that was a useful thing to do?

Female: I don't know because I don't think we got any feedback on what actually happened in the end, because it was over a long period. I don't think we did, did we, *** ? [So not to get many back, no].

Facilitator 2: Okay so if we move to your perceptions of the ECOH program. So if you think about the program, the whole process including Lift the Lip, referral process, documentation. How do you feel about the program? What are the positive things about it? What are negative things about it perhaps? What could be improved?

Female: To be honest, I don't hear one thing that stands out in my mind - a positive or a negative about it so I can't give you any feedback.

Female: No me neither.

Female: Does anyone else?

Facilitator 2: So it's - you think it's a worthwhile program? Do you think it's working?

Female: Compared to what?

Female: Yeah the thing is they don't come to us for their children's teeth. They come to us for a whole range of things so we don't focus just on their teeth. We focus on their overall health, their development. Sometimes what's happening in the family, the dynamics of the family and what's happening with mum? Is she okay or if she's not okay, baby's not going to be okay, all those sorts of things. We don't think so and so is coming and he's 18 months and we've got to make sure that that mum's doing the teeth brushing because there might be other things that he's falling behind in. So that's my thought anyway.

Facilitator: Have you had instances where oral has not been an issue. So that may be one of the reasons. It doesn't jump out.

Female: As *** is saying, when we discuss solids with them, you tend to see who's going to be feeding their child well and who's not because they're telling you they're only shoving chocolate in the baby's hand when their just starting solids kind of thing. So it's educating the parents definitely. You can't really say.

Facilitator: No but if having a pathway does it make you guys feel more empowered to do something for the child or if you didn't have it and you could just say please go to a dentist, would that be the same?

Female: We tend - if we have a pathway for anything we generally follow it.

Facilitator: Yeah because ECOH basically gives you guys a pathway to send the child for a free treatment so if that barrier is removed?

Female: If we go through referral and then obviously you guys will contact the parents.

Facilitator: Yes.

Female: So that's the best for them to do as well. Sometimes they've got so much stuff to do and they just forget about it or something. So by you doing it, it feels like you've done something for them and it's easier that way. So that's one thing that they're definitely going to get a follow up and we'll be seeing where if they leave the clinic and you don't know whether the mum will call and will go to the dentist or not. So at least it reassures you that the child's going to be looked at.

Facilitator 2: Are there any barriers that you're aware of in terms of parents accessing dental services or coming in?

Female: No.

Facilitator: Do you feel have any mother discuss why they've not been able to take their child or don't think that's important to take the child.

Female: [Didn't have any issues so, no].

Facilitator: Yeah doesn’t come, hasn't come.

Female: No.

Facilitator: That's good.

Facilitator 2: Okay so we'll move to training. So did you receive training to undertake ECOH? So you've all had training.

Female: Yep.

Facilitator 2: Okay when was the last time you had training?

Female: About two years ago.

Female: Probably two years yeah.

Facilitator 2: Okay so do you think it needs to be reviewed?

Female: Yep.

Facilitator 2: How often would you think it should be reviewed?

Female: Every year.

Female: Every year.

Facilitator 2: Okay and would you expand it? Are there any changes you think you'd make to the training or suggest?

Facilitator: The main information provided in the training is sufficient for you too.

Female: Yeah we do the Lift the Lip and all those things with the babies anyway when they come. Some things are the same.

Facilitator: So what mix of people come to you, to this part? Is it multicultural mainly? What particular ethnic group, a lot of Aboriginal children?

Female: Everything.

Female: We do have an Aboriginal program so yeah. They're not too bad.

Female: I've got Aboriginal mums and there's no problems with their kids. They do go to paediatrician clinics and everything. We check them. I haven't had any issues with their kids. We teach them from the beginning.

Facilitator: So their general and oral health is similar to non-Aboriginal child.

Female: Yeah.

Facilitator: That's really good.

Female: I think the Aboriginal pamphlets are quite - when the parents see that, they get a bit more shocked.

Facilitator: So that black shocking teeth it's a bit confronting for them.

Facilitator 2: Are there any extra resources that you think would make your job a bit more easier?

Female: I do actually because in the waiting rooms for the clinics, I think it's an ideal opportunity to educate. I do that swimming, you know the *** one. Pool safety and water safety and stuff like that. I think if you just had a five minute DVD on dental health for babies and for children that we could just run over and over, out here would be an ideal opportunity. That's my point of view because you get such a cross section of people, antenatal, the whole lot. Instead of watching the whatever, look at some educational - that's what I think.

Facilitator 2: Rather than watching TV.

Female: Rather than watching the cricket.

[Laughter]

Facilitator: If we sent out a DVD to you, would you be able to run it?

Female: Yep.

Female: Yeah definitely.

Facilitator: Because we do have DVDs that have [unclear] and they're not - yes children is fine, but it's also about the parents and about immigrants, or in a multicultural group, there's a DVD that was shot. I think it's about a 10 minute DVD and it talks about how - it's done in a skit format where a girl, she's going on a debate team and she's very - she doesn't care about her teeth and her mum's always taking. There's another immigrant family and the wife wants to avoid children doing certain things but the dad makes - oh that's fine it doesn't matter. There's a conflict there so they're different scenarios and they talk oral

health. So it's available on the website to download as well but I'm happy to send you a DVD.

[Over speaking]

Facilitator: There's one by *** just for kids, young kids. It's called *Doctor Tooth*.

Female: What they don't know won't do them any harm.

Female: I just think it's an ideal opportunity.

Facilitator 2: So information in community health centres, doctor's surgeries. You said pre-school, playgroup perhaps.

Female: Yeah day care centres.

Facilitator 2: Any other places that you can think of?

Female: Antenatal clinics.

Facilitator 2: Antenatal clinic yeah. So something like the DVDs and literature as well.

Female: Yep.

Facilitator 2: If you were going to design it, they just said okay you can design whatever you like around oral health, have all the resources you want. What would you ask for?

Female: Free toothbrushes for kids.

Female: Definitely.

Female: At an early age so that they can play with them at a very early age to get things going into their mouth, to explore, to develop. One toothbrush to each baby I reckon.

Facilitator 2: Sounds cheap.

Female: …and a Blue Book.

[Laughter]

Facilitator 2: …and the Blue Book.

Female: There was talk about years and years ago where they were going to do that but nothing ever came of it.

Female: It's a shame isn't it? Take the place of a dummy and give them a toothbrush.

Female: They were going to give toothbrush…

Female: Training cup or something.

Female: Yeah they were going to give them a training cup and then they were going to give them a toothbrush at 12 months or something, toothpaste and then nothing. It all fell apart.

Female: That's your mission.

[Laughter]

[Over speaking]

Female: Write to the minister.

Facilitator: Comes back to the cost and the sustainability because there has to be a pattern of who's going to deliver it. Where it has to be delivered, how often? Is it being misused? Is it being really used? Is there a long term effect of giving it or not. So I think there's a project happening in - even in South West where they're just sending out the brushes and these mum's were recruited when they gave birth. So they got recruited into the study and at six months they got information.

Then at 12 months they got the sippy cup. At 18 months they started getting a brush and every two years now they get a brush and a paste every six months to see when they come at five years if there's any change in oral health of this particular group [of patients].

To see if there's really anything or the brush comes and someone else takes it and it never goes to the child then, stuff like that.

Female: Or they brush the dog's teeth.

[Laughter]

Female: Is there a brochure around for - and I was thinking about the clinics for when a baby is first born until say two - on how to start introducing a toothbrush or getting things into their mouth and things like that.

Facilitator: Yes there are plenty of brochures. There's about how to encourage them to drink from a cup. There's also how to, when to brush for a child. How to brush, what is action? How should they be shown and the actions.

Female: I haven't seen that.

Female: No I haven't.

Female: We'd be interested.

Female: I saw some brochures but there's none of them in there because I was putting them all away.

Female: Last Friday I was [unclear].

[Over speaking]

Female: When and where do I do this? When do I start dah, dah.

Female: I think you can buy the three year applicators in a pack. They're put out by a company, I can't think which one, where it starts off with they've got a guard on so they can't actually put them too far down. One of the mum's brought them into the clinic and I thought what a jolly good idea they were. Poke an eye out probably.

[Laughter]

Facilitator 2: They were talking about kids getting locked in cars because the parents give them the car keys - to the child to chew on while they're…

Female: Oh really?

Facilitator: Yeah so substitute keys for the toothbrush.

Female: Yeah they do give them keys to play with [unclear] in their mouth.

Female: Just think about all the germs.

Female: I'm thinking about the [car, and] the saliva going into the…

[Laughter]

Female: My son started chewing on the iPhone and slowly the sound disappeared.

[Laughter]

[Aside discussion]

Facilitator: The brochure [of] kids under five - there's a brochure and that has about all the things for kids under five.

Female: We'd love some.

Facilitator: Okay so DVD and kids under five. Any other requests?

[Over speaking]

Facilitator: Another training organised as well. If you'd like another training, you just have to send a request because *** is the co-ordinator and normally who wants the training, just send an email to her and she comes out and does the training or one of the dental therapists come out.

Facilitator 2: Okay so are there any other issues that you'd like to talk about?

Facilitator: Jog your memories.

Facilitator 2: Any great stories [laughs].

Facilitator: Good. Thank you for your time and sharing your ideas and opinions. Really appreciate it and hopefully we can improve the system and hopefully you guys see no more of people with black teeth. Keep your record going. Excellent, thank you very much.

END OF TRANSCRIPT
